# The Dawn of Single Material Organic Solar Cells

**DOI:** 10.1002/advs.201801026

**Published:** 2018-10-09

**Authors:** Jean Roncali, Ion Grosu

**Affiliations:** ^1^ Group Linear Conjugated Systems Moltech Anjou CNRS University of Angers 2Bd lavoisier 49045 Angers France; ^2^ Supramolecular Organic and Organometallic Chemistry Center Babeş‐Bolyai University 11 Arany Janos str. 400028 Cluj‐Napoca Romania

**Keywords:** acceptors, donors, excitons, organic solar cells

## Abstract

Single material organic solar cells (SMOSCs) are based on ambivalent materials containing electron donor (D) and acceptor (A) units capable to ensure the basic functions of light absorption, exciton dissociation, and charge transport. Compared to bicomponent bulk heterojunctions, SMOSCs present several major advantages such as considerable simplification of cell fabrication and a strong stabilization of the morphology of the D/A interface, and thus of the cell lifetime. In addition to these technical issues, SMOSCs pose fundamental questions regarding the possible formation, and dissociation of excitons on the same molecular D–A architecture. SMOSCs are developed with various approaches, namely “double‐cable” polymers, block copolymers, oligomers, and molecules that differ by the donor platform: polymer or molecule, the nature of A, the D–A connection, and the intra‐ and intermolecular interactions of D and A. Although for several years the maximum efficiency of SMOSCs has remained limited to 1.0–1.5%, impressive progress has been recently accomplished leading to SMOSCs with 4.0–5.0% efficiency. Here, recent advances in the synthesis of D–A materials for SMOSCs are presented in the broader context of the chemistry of organic photovoltaic materials in order to discuss possible directions for future research.

## 1 Introduction

Solar energy is expected to provide a major contribution to an increasing energy demand in a context of reduction of the carbon emission associated with the use of fossil energy sources. In this context, the photovoltaic (PV) conversion of solar light into electricity is an area of intense research activity. Although the industry of solar PV modules based on silicon solar cells is well established, research on alternative PV technologies has been pursued for several decades.^1^ In this regard, organic photovoltaic (OPV) cells are potentially attractive because of a unique combination of properties: lightweight, flexibility, plasticity, low environmental impact, and low cost. Research on OPV was initiated in the mid‐seventies, in the wake of earlier basic research on the photovoltaic properties of organic conjugated systems.^2^, ^3^, ^4^ These first OPV cells basedon dyes or pigments which had not been specifically designed for PV conversion were poorly efficient with power conversion efficiencies (PCEs) of ≈0.01–0.10%.^5^ The fabrication of the first organic heterojunction by contacting an electron donor material (D) with an electron acceptor (A) by Tang in 1986 represents a milestone in OPV research.^6^ Due to the high electric field generated at the D/A interface, excitons diffusing to the interfacial zone are efficiently dissociated, allowing for the first time, the PCE to reach values close to 1.0%.^6^ Today, planar heterojunction (PHJ) cells essentially fabricated by vacuum deposition are still investigated for both technological and basic research and remain an invaluable tool for the screening of new active materials.^7^, ^8^, ^9^, ^10^, ^11^

Due to the high dielectric constant of silicon (ε_R_), photon absorption by a silicon solar cell leads to delocalized excitons of low binding energies (5–15 meV) which are easily thermally dissociated at ambient temperature. In contrast, because of the low dielectric constant of organic semiconductors, light absorption by an OPV cell generates Frenkel excitons, i.e., hole–electron pairs tightly bounded by strong Coulombic attraction with binding energies of several hundreds of millielectronvolts which require high electric field to dissociate.^12^, ^13^ Based on this specific process, the term of “excitonic solar cells” has been proposed to qualify OPV cells as well as dye‐sensitized solar cells (DSSCs).^14^ The short diffusion length of excitons in organic materials (typically 10–20 nm) imposes a drastic limitation to the efficiency of OPV since it imposes an equivalent limit to the maximum thickness of the active layer, which in turns limits the amount of absorbed photons and hence PCE.

A new wave of research on OPV was initiated by the encounter of two classes of materials, namely π‐conjugated polymers,^15^ and fullerenes.^16^, ^17^, ^18^, ^19^ The discovery of a fast and efficient photoinduced electron transfer between poly(*p*‐phenylenevinylene) (PPV) and fullerene C_60_ reported in 1992 by Sariciftci et al.^20^ and the subsequent invention of the bulk heterojunction (BHJ) solar cell^21^, ^22^ have impulsed an intense research effort on OPV.

**Scheme 1:**
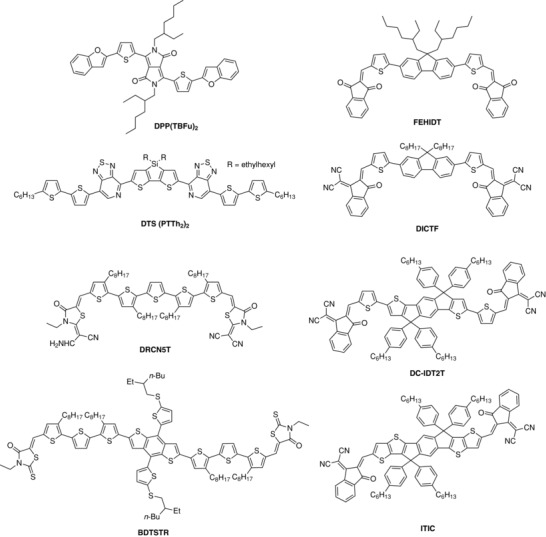
Chemical structure of examples of highly efficient molecular donors (left side) and acceptors (right side). DPP(TBFu)_2_,^39^ DTS(PTTh_2_)_2_,^41^ DRCN5T,^42^ BDTSTR,^43^ FEHIDT,^44^ DICTF,^45^ DC‐IDT2T,^46^ ITIC.^47^

BHJ cells are fabricated at ambient temperature by processing blend films from formulated solutions of D and A.^23^, ^24^ Solvent evaporation produces a biphasic film in which D and A domains should form two independent continuous networks. A high quantitative quantum efficiency of photon/electron conversion requires the control of the size and continuity of the D and A domains to dimensions commensurate with the exciton diffusion length while providing percolation pathways for the photogenerated positive and negative charges. The control of the nanoscale morphology of the interface is the key to the fabrication of BHJs.^25^ This problem, investigated in hundreds of publications, is of course tightly related to the nature, physical, chemical, and electronic properties of the active materials. Conjugated polymers have been for a long time the unique class of D materials for BHJs. PPV derivatives used in early work^21^, ^22^ were supplanted by poly(3‐hexylthiophene) (P3HT) which became a standard donor material,^26^ and more recently by new generations of low bandgap polymers.^27^, ^28^, ^29^ On the other hand, soluble derivatives of fullerenes C_60_ and C_70_, namely [6,6]‐phenyl‐C_61_‐butyric acid methyl ester (PC_61_BM) and PC_71_BM have been the standard acceptor materials for many years.^23^, ^24^, ^25^ Based on the advantages of reproducible synthesis, purification, and properties, soluble conjugated molecules have been proposed in 2006 as alternative donor materials to polymers.^30^, ^31^ In the past decade, the chemistry of molecular donors has rapidly expanded and hundreds of new molecules have been synthesized and evaluated.^32^, ^33^, ^34^, ^35^, ^36^, ^37^, ^38^, ^39^ Whereas the first examples of molecular donor such as thiophene or PPV oligomers or acenes possess a homogeneous electronic structure,^38^ molecular donors of second generation are based on combinations of D and A building blocks in order to create an internal charge transfer which at the same time improves the light‐harvesting properties of the material and increases the cell voltage.^40^ In recent years, several classes of donors of symmetrical structure, as exemplified in **Scheme** **1**, have led to BHJs with PCE comparable to those of the best polymer cells.^36^

Owing to high electron mobility and isotropic charge transport, fullerenes derivatives have become the standard acceptor materials for OPV. However, fullerenes also present some drawbacks such as a limited absorption of visible light and the difficult modulation of their electronic properties. Research on nonfullerene acceptors (NFAs) was initiated some years ago,^48^ but in the past two years, the topic has evolved extremely fast and some NFAs based on A and D building blocks (Scheme 1) have reached performances surpassing those of fullerenes derivatives.^43^, ^45^, ^46^, ^47^, ^49^, ^50^, ^51^ The emergence of these new NFAs has also changed the vision of OPV cells by drawing more attention to the contribution of the acceptor to the absorption of incident light and to the process of hole transfer from A to D.^52^

The optimization of a BHJ cell is an extremely complex task involving due mutiple experimental parameters such as cell architecture (direct or inverted structure) formulation of the D+A feeding solution, solvent, method and conditions of film processing, insertion of buffer layers, bimetallic electrodes (Al+Ca or Ba), additives, thermal treatments, and solvent annealing.^23^, ^24^, ^25^, ^53^ All of these highly critical parameters have been intensively investigated, giving rise to hundreds of publications. During the past two decades, intensive multidisciplinary research effort has produced spectacular progress with the PCE of BHJ cells increasing from 1.0% to 13–15% in 20 years.^54^, ^55^, ^56^ The control of the extension, structure, and properties of the D/A interfacial zone is the key to the performances of BHJ cells. In particular, much effort has been directed toward the control of the nanophase separation of D and A in blend films by means of different techniques involving the formulation of the feed solution, the application of physical processes, or the use of additives.^23^, ^24^, ^25^, ^26^, ^57^ However, a major problem of biphasic BHJs is that the optimized morphology is thermodynamically unstable and the progressive macrophase separation of D and A leads to a decrease of PCE.^58^, ^59^

In this context, the fabrication of OPV cells using only one material can present several decisive advantages. From a technical point of view, single material organic solar cells (SMOSCs) will considerably reduce the complexity and cost of cell fabrication while providing a possible definitive solution to the morphological instability of multicomponent BHJs.^60^ The synthesis of active materials for SMOSCs implies the association of D and A parts into molecular architectures capable to achieve the elemental processes of light absorption, exciton dissociation, and charge transport usually ensured by two different materials in BHJs. In spite of these major potential advantages, SMOSCs have, until now, attracted much less attention than BHJs. This situation may be related to the considerable difficulty of the task and to the widespread idea that because of fast charge recombination and inefficient charge hopping and transport, SMOSCs cannot reach high PCEs.^34^

**Figure 1:**
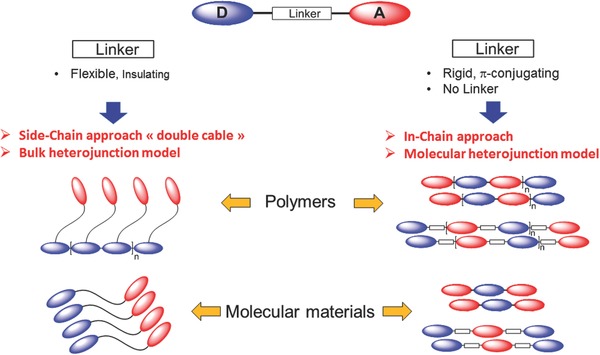
Synthetic approaches for the development of polymeric and molecular photoactive materials for SMOSCs.

The design and synthesis of active materials for SMOSCs is a complex multiparameter problem in which optimal absorption of solar light, controlled energy levels of D and A, charge separation, and charge transport must be simultaneously taken into account. Although the single layer Schottky diodes investigated during the seventies^5^ can be viewed as the ancestors of SMOSCs, the first prototypes of cells specifically designed as SMOSCs appeared only after the invention of BHJs and were largely inspired by the BHJ model.^60^ The fabrication of SMOSCs can be envisioned in different ways that differ essentially by the nature of D (polymer or molecule) and A (fullerene or nonfullerene) and before all, by their mode of connection. In a first approach based on the two‐component BHJ paradigm, D and A are covalently linked by a flexible insulating spacer (L) long enough to allow the self‐organization of D and A into separated domains (**Figure** **1**). In this case, the nature and length of the flexible linker are determining factors for the morphology and nanophase separation of D and A in the material. More generally and although thermal treatments and additives can be effective, the morphology of the active layer of SMOSCs depends primarily on the chemical structure of the active material. Whereas in such D–L–A systems, excitons are dissociated at the interface of D and A domains, a different approach based on the molecular heterojunction model^61^, ^62^, ^63^ reminiscent of DSSCs,^64^ aims at confining the charge‐separation process at a D/A interface localized on the same molecular architecture (Figure 1).

In fact, the possible creation and dissociation of excitons on the same molecular site poses fundamental questions which could have major implications regarding the limitations imposed by the short exciton diffusion length to the efficiency of OPV cells. The process of photoinduced charge and/or energy transfer in D–A dyads has given rise to the synthesis of huge number of molecular architectures and to numerous photophysical studies.^61^, ^62^, ^63^ However, these systems were essentially designed in a frame of molecular chemistry, and extending this concept to SMOSCs implies that the design of D–A systems takes also into account the molecular organization of the material and in particular the transport of electrons and holes.

Research on SMOSCs has recently witnessed important progress on both polymeric and molecular materials and the best PCE which has remained around 1.50% for several years,^60^ has now increased up to values of ≈4.0–5.0% with the highest PCE of 5.58%.^65^ The aim of this short review is this to discuss recent advances in this emerging field in order to identify possible directions for future work and stimulate further research in this promising and exciting area.

## 2 SMOSCs Based on Polymeric Active Materials

In the past few years, research on polymer‐based SMOSCs has been focused on two types of materials, namely polymers or copolymers with pendant acceptor groups and “in‐chain” systems in which the acceptor is directly inserted in the polymer backbone.

### 2.1 “Double‐Cable” Polymers with Fullerene Acceptor Groups

The convergence of the development of BHJ solar cells with the emergence of the chemistry of fullerenes at the end of the nineties has led to the synthesis of conjugated polymers with fullerenes (essentially C_60_ derivatives) attached on the π‐conjugated backbone by flexible linkers.^60^, ^61^, ^62^, ^63^, ^66^ Cravino and Sariciftci have proposed the concept of “double‐cable” polymers in which the donor is constituted by a π‐conjugated polymer backbone which is expected to transport positive charges, while pendant C_60_ groups attached by a flexible spacer should ensure the transport of electrons.^66^ In fact, such a view appears as a bit simplistic since in a 3D material electrical charges do not necessarily follow these assigned tracks but are transported by a hopping mechanism between neighboring chains or mole‐cules. Although a number of such C_60_‐based “double‐cable” polymers have been synthesized, only a few of them have been evaluated as active materials in OPV cells and an even smaller number has demonstrated effective photovoltaic conversion properties. Conjugated polymers with pendant C_60_ units can be synthesized by various approaches such as the chemical or electrochemical polymerization of a precursor bearing covalently attached C_60_, the fixation of C_60_ onto a preformed polymer containing ω‐bromoalkyl reactive groups, or the synthesis of block copolymers.^60^, ^61^, ^62^, ^63^, ^66^

**Scheme 2:**
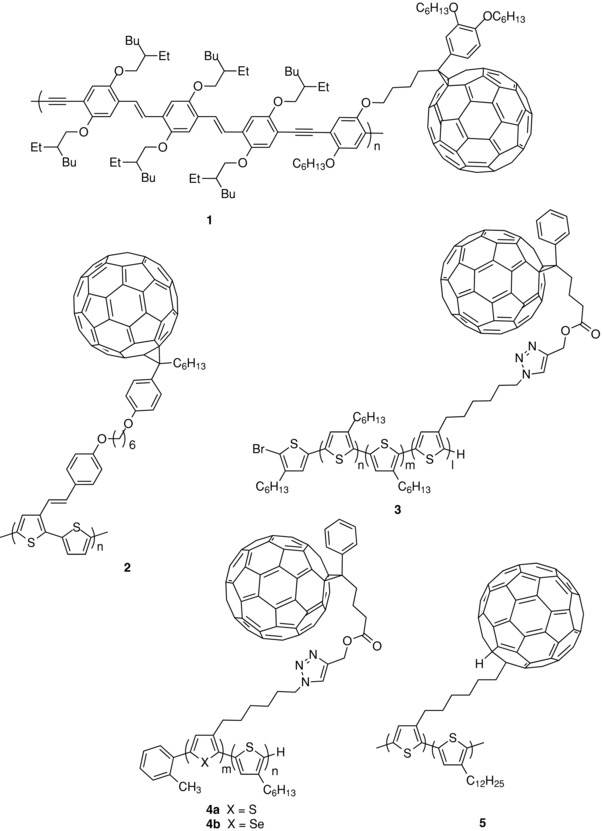
Chemical structure of polymers **1**–**5**.

Already in 2001, Zhang et al. reported the copolymerization of a C_60_‐substituted thiophene monomer with a monomer unit bearing a solubilizing polyether chain. The C_60_ content of the final copolymer was adjusted by controlling the ratio of the two monomers. The resulting photodiode gave an efficiency of 0.60% under 505 nm monochromatic light.^67^ Marcos Ramos et al. synthesized a C_60_‐derivatized copolymer (**1**) (**Scheme** **2**), by reaction of a di‐iodobenzene bearing an attached C_60_ with oligo‐phenylenevinylenes (*n*PVs) possessing a terminal alkyne. Under white light irradiation at 100 mW cm^−2^, a cell of structure ITO/PEDOT:PSS/**1**/aluminum delivered a short‐circuit current density (*J*_sc_) of 0.42 mA cm^−2^, an open‐circuit voltage (*V*_oc_ ) of 0.83 V, and a fill‐factor (FF) of 0.29, leading to a PCE of 0.10%^68^ (**Table** **1**). In 2007, Li and co‐workers reported a “double‐cable” polymer obtained by postpolymerization fixation of C_60_ onto a polymer prepared by polymerization of a bithiophenic precursor (**2**). A cell of structure of indium tin oxide (ITO)/poly(3,4‐ethylenedioxythiophene):polystyrene sulfonate (PEDOT:PSS)/**2**/Ca/Al of 4 mm^2^ active area gave a *J*_sc_ of 2.41 mA cm^−2^ and a PCE of 0.52% under AM 1.5 simulated solar light.^69^ Three years later, Tajima and co‐workers reported the synthesis of poly(3‐alkylthiophene)‐based diblock copolymers with attached C_60_ groups (**3**). Diblock copolymers of 3‐hexylthiophene and 3‐(6‐bromohexyl)‐thiophene were synthesized by Ni‐catalyzed quasi‐living polymerization, leading to polymers with a number‐averaged molecular weight (*M*_n_) of 24.3 kDa and a polydispersity index (PDI) lower than 1.1.^70^ The terminal bromides at the side chains were then quantitatively converted into azides by NaN_3_ and a fullerene bearing an alkyne group was then attached to the side chain by a “click” cycloaddition reaction. After thermal annealing at 130 °C, a SMOSC based on copolymer **3** delivered a *J*_sc_ of 6.15 mA cm^−2^, a *V*_oc_ of 0.54 V, and a FF of 0.51, leading to a PCE of 1.70%. The external quantum efficiency (EQE) spectrum showed a maximum of 49% at 515 nm. Comparison with a classical BHJ cell based on P3HT and PC_61_BM showed that in spite of a higher initial PCE of 3.0%, the BHJ cell undergoes a much faster degradation under prolonged thermal treatment than the SMOSC.^70^ In further investigations of the synthesis and structure of the diblock copolymers, the same authors have synthesized P3HT‐based diblock copolymers with controllable block lengths by combining Grignard metathesis and nickel‐catalyzed quasiliving polymerization.^71^ As in their previous report, the final fixation of C_60_ was quantitatively achieved by click chemistry. The UV–vis absorption spectra of the copolymers in chloroform were practically identical to that of a 1.0:0.6 weight ratio of a mixture of P3HT:PC_61_BM. A significant fluorescence quenching of P3HT was observed for diluted solutions of the copolymers, while this phenomenon was not observed for mixtures of the two components at similar concentrations of P3HT. Solar cells of structure ITO/PEDOT:PSS/active layer/Al were fabricated and characterized under AM 1.5 simulated solar light. The best results were obtained with a copolymer containing a block of regioregular P3HT, which gave after optimization a PCE of 2.46% with a *J*_sc_ of 8.14 mA cm^−2^, a *V*_oc_ of 0.48 V, and a FF of 0.63. These values are rather close to those obtained with a reference classical P3HT:PC_61_BM BHJ cell (*J*_sc_ = 8.61 mA cm^−2^, *V*_oc_ = 0.54 V, FF = 0.66, and PCE = 3.07%.^71^ More recently, the processes of charge generation and recombination in the block copolymers were investigated by transient absorption spectroscopy using a bicomponent PH3T/PC_61_BM BHJ as reference. It was found that in disordered domains, 35% of P3HT polarons geminately recombine to the ground state in P3HT–PC_61_BM copolymer films, while no geminate recombination is observed in P3HT/PC_61_BM blend films. This higher geminate recombination was attributed to micelle‐like defects in microphase‐separated structures. In contrast, such loss was found negligible upon excitation at 500 nm of the crystalline domains of the copolymer. Based on the crystallinity of the films (≈40%), it was concluded that the overall charge generation loss due to recombination was 20% at most. On the other hand, the charge‐carrier lifetime was longer in P3HT–PC_61_BM copoly‐mer films than in P3HT/PC_61_BM blend films.^72^ In a more recent work, the same group reported the synthesis of heteroblock copolymers consisting of P3HT and fullerene‐attached poly(3‐alkylselenophene) (**4b**).^73^ The idea was to create a cascade of energy levels in order to limit charge recombination. The copolymers were obtained by quasiliving catalyst transfer polycondensation and subsequent conversion reactions. An *o*‐tolyl‐substituted nickel catalyst was used instead of Ni(dppp)Cl_2_ because of its high initiation efficiency and because propagation from both ends of the polymer chain could be avoided. Characterization of the polymers confirmed the formation of well‐defined diblock structures with high loading of fullerene at the side chain (≈40 wt%). After thermal annealing, solution‐cast thin films of the copolymers showed a clear microphase‐separated nanostructure of ≈30 nm in repeating unit, identical to the microphase‐separated nanostructure of diblock copolymer of P3HT and fullerene derivatized poly(3‐alkylthiophene). OPV cells of inverted structure ITO/ZnO/**4**/MoO_3_/Ag of 12 mm^2^ active area were fabricated and characterized under AM 1.5 simulated solar light (100 mW cm^−2^). Devices fabricated from the as‐cast films showed negligible photovoltaic conversion for both **4a** and **4b**, possibly due to poor connection of the fullerene domains in the copolymer, as suggested by transmission electron microscopy (TEM) and atomic force microscopy (AFM) images. After thermal treatment at 250 °C, the best cells made with both the copolymers gave comparable performances, namely PCE = 1.38% for the thiophene‐based copolymers and 1.52% for the selenophene‐based copolymers.^73^

**Table 1:** Cell structure and photovoltaic characteristics of SMOSCs based on polymeric photoactive materials under simulated AM 1.5 solar light. PL: photoactive layer; aa: active area; SA: standard anode = ITO/PEDOT:PSS; nr: not reported

Compound	Cell structure	aa [mm^2^]	EQE_max_ a)	*J*_sc_ [mA cm^−2^]	*V*_oc_ [V]	FF	PCE [%]	Ref.
**1**	SA/PL/Al	nr	nr	0.42	0.83	0.29	0.10	68
**2**	SA/PL/Ca/Al	4	nr	2.41	0.75	0.29	0.52	69
**3**	nr	nr	0.49	6.15	0.54	0.51	1.70	70
**3**	SA/PL//Al	6	0.56	8.14	0.48	0.63	2.46	71
**4a**	ITO/ZnO/PL/MoO_3_/Ag	12	0.38	4.81	0.51	0.57	1.38	73
**4b**	ITO/ZnO/PL/MoO_3_/Ag	12	0.41	5.02	0.51	0.59	1.52	73
**5**	SA/PL/Al	6.25	0.52	10.7	0.59	0.55	3.47	65
**5**	SA/PL/Al	6.25	0.54	13.4	0.68	0.62	5.58	65
**6b**	SA/PL/Al	nr	0.31	1.50	0.44	0.25	0.20	75
**7**	SA/PL/LiF/Al	6	nr	2.57	0.51	0.37	0.49	78
**8**	SA/PL/LiF/Al	6	0.45	4.30	0.57	0.34	0.89	79
**9a**	ITO/ZnO/PL/MoO_3_/Ag	4	0.10	3.21	0.54	0.30	0.51	80
**9b**	ITO/ZnO/PL/MoO_3_/Ag	4	0.25	7.03	0.57	0.41	1.64	80
**9c**	ITO/ZnO/PL/MoO_3_/Ag	4	0.35	8.96	0.68	0.45	2.74	80
**10a**	ITO/ZnO/PL/MoO_3_/Ag	4	0.64	7.54	0.69	0.53	2.72	82
**10b**	ITO/ZnO/PL/MoO_3_/Ag	4	0.67	8.05	0.78	0.57	3.60	82
**10c**	ITO/ZnO/PL/MoO_3_/Ag	4	0.66	7.60	0.92	0.60	4.18	82
**11**	SA/PL/Al	16.2	0.35	5.20	1.23	0.47	3.10	84
**12**	SA/PL/Al	16.2	–	0.36	0.58	0.27	<0.1	86
**13**	SA/PL/Al	16.2	–	4.73	1.11	0.42	2.24	87
**14a**	SA/PL/Al	16.2	–	0.07	0.14	0.28	0.003	88
**14b**	SA/PL/Al	16.2	0.09	1.33	1.12	0.37	0.55	88
**14c**	SA/PL/Al	16.2	0.17	1.95	1.08	0.45	0.95	88
**15**	–	–	nr	0.13	0.52	0.36	0.025	89
**16a**	ITO/ZnO/PL/MoO_3_/Ag	4	0.10	1.30	0.79	0.35	0.36	90
**16b**	ITO/ZnO/PL/MoO_3_/Ag	4	0.25	4.04	0.79	0.48	1.54	90
**17a**	SA/PL/Ca/Al	14	–	0.06	0.59	0.25	0.01	91
**17b**	SA/PL/Ca/Al	14	0.26	2.42	0.33	0.28	0.22	91
**17c**	SA/PL/Ca/Al	14	0.03	0.36	0.21	0.23	0.02	91
**18**	ITO/ZnO/PL/MoO_3_/Ag	6	0.52	8.30	0.93	0.50	3.87	92
**19**	SA/PL/Ca/Al	12	–	1.80	0.61	0.28	0.30	93
**20**	SA/PL/Ca/Al	–	0.37	5.29	0.43	0.43	1.00	95

^a)^ Taken at the maximum of the visible spectral region.

Very recently, Pierini et al. have reported the synthesis of nanofibers of polythiophene with attached C_60_ (**5**) as active material for SMOSC.^65^ A copolymer of 3‐dodecylthiophene and 3‐(bromohexylthiophene) was synthesized by Grignard metathesis (GRIM) followed by nickel‐catalyzed polymerization. The grafting of C_60_ was then achieved by reacting the Grignard reagent of the precursor copolymer in tetrahydrofuran (THF) with C_60_ in toluene. ^1^H NMR spectrometric data indicated a regioregularity of 95% for the precursor polymer and a 0.55:0.45 molar ratio for 3‐dodecylthiophene and 3‐(bromohexylthiophene). A lower ratio of fullerene‐substituted 3‐hexylthiophene was expected in the final polymer due to the nonquantitative yield of the substitution reaction. The ratio of 3‐dodecylthiophene and 3‐(bromohexylthiophene) used for the synthesis of the copolymer has been optimized on the basis of the PCE of the SMOSCs fabricated with the various copolymers. It was found that PCE increases from 2.58% for an 85:15 ratio to 2.90% for 70:30, while the best results (PCE = 3.47%) were obtained for a 58:42 ratio. This ratio appeared to be the upper limit since a 50:50 ratio led to problems of solubility and processability. It is worth noting that a PCE of ≈3.50% represents the average value of the standard bicomponent BHJ cells fabricated with P3HT and PC_61_BM.^26^ Nanofibers of the copolymer were obtained by electrospinning a chloroform solution of the copolymer containing 0.7% of poly(ethyleneoxide) (PEO). After removal of PEO, the X‐ray diffraction data of the fibers reveal the presence of a (010) peak at 2θ = 23.5°, typical of π‐stacking. The fact that this peak is absent in the diffractogram of solution‐cast films but present in that of the fibers indicates that the electrospinning process induces an alignment of the polymer chains which promotes interchain π‐stacking and crystallization. A dispersion of the nanofibers in cyclohexanone was deposited on an ITO/PEDOT:PSS electrode and recovered by a layer of fully solubilized copolymer in order to ensure the interconnection of the nanofibers.^74^ Under AM 1.5 simulated solar light, a SMOSC of 6.25 mm^2^ based on **5** gives after thermal treatment a PCE of 3.50%, similar to that obtained with a reference two‐component BHJ cell P3HT–PC_61_BM. On the other hand, the best cell based on the nanofibers reaches a PCE of 5.58%, which is at present the highest value reported for a SMOSC, even surpassing the best results obtained on BHJs based on the P3HT/PC_61_BM D/A pair.^65^ Stability tests in ambient conditions have shown that this cell still presents a PCE of 4.62% after 42 days of storage. Although the scaling up of this technology to industrial production might be problematic, from a fundamental point of view, this remarkable result clearly demonstrates that contrary to a rather common opinion, SMOSCs can reach efficiencies comparable or even better than those of multicomponent devices based on the same D/A pair.

### 2.2 “Double‐Cable” Polymers with Nonfullerene Acceptor Units

**Scheme 3:**
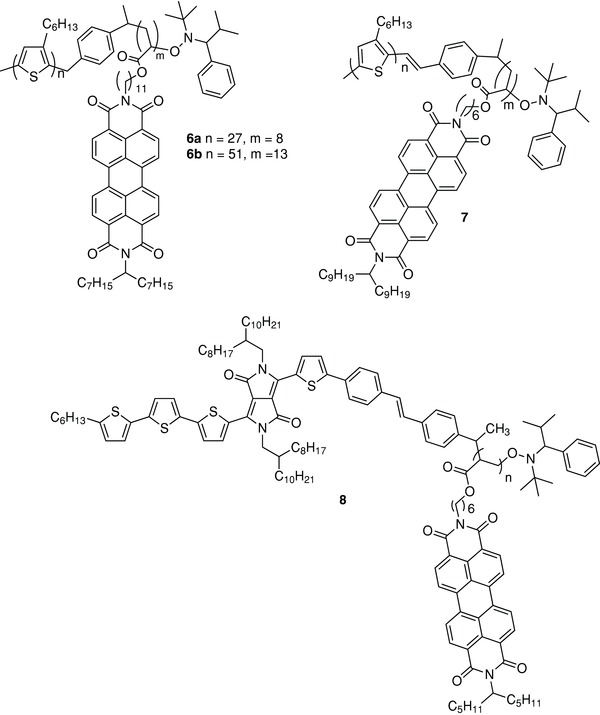
Chemical structure of polymers **6**–**8**.

The fabrication of SMOSCs based on block copolymers combining π‐conjugated blocks and nonfullerene acceptors has been reported by several groups. Thus, Thelakkat and co‐workers have extensively investigated several types of donor–acceptor diblock polymers using nitroxide‐mediated polymerization.^76^, ^77^ They have shown that a copolymer based on P3HT as donor block and perylene bisimide (PBI) acrylate as acceptor (**6**) (**Scheme** **3**) presents specific crystallization of the two domains, as confirmed by the observation of two melting points and by scanning electron microscopy (SEM) images that revealed the presence of microphase‐separated domains.^77^

The quenching of the photoluminescence was consistent with a photoinduced electron transfer from P3HT to the PBI acceptor. The influence of the molecular weight (MW) was analyzed on two copolymers of MW of 16.1 kDa (**6a**) and 29.5 kDa (**6b**). Copolymer **6b** showed an ≈2 orders of magnitude higher hole mobility than **6a**. SMOSCs of structure ITO/PEDOT:PSS/**6a** or **6b**/aluminum based on spun‐cast films of the copolymers gave a maximum EQE of 31% at 495 nm for **6b** and only 3% for **6a**. The best device gave a *J*_sc_ of 1.50 mA cm^−2^, a *V*_oc_ of 0.44 V, and a PCE of 0.20%.^75^ A parent diblock copolymer involving regioregular P3HT and PBI acrylate (**7**) was reported by Zhang et al.^78^ A SMOSC consisting of ITO/PEDOT:PSS/ **7**/LiF/Al, gave a *J*_sc_ of 0.67 mA cm^−2^ and a PCE of 0.11% under AM 1.5 simulated solar illumination at 100 mW cm^−2^. Thermal treatment at 150 °C for 20 min increased *J*_sc_ to 2.57 mA cm^−2^ and PCE to 0.49%.^78^

Koyuncu et al. reported the synthesis of a highly crystalline D–A low bandgap polymer with oligothiophene–diketopyrrolopyrrole (DPP) as the donor and PBI as the acceptor (**8**) using a DPP‐based macroinitiator and acryloyl‐based PBI.^79^ The degree of polymerization estimated from ^1^H NMR data was limited to 3–4 units. The UV–vis absorption spectrum of dichloromethane solutions presents a broad absorption in the visible and near infrared (NIR) region in which the DPP macroinitiator and PBI exhibit complementary absorptions. The absorption edge at ≈750 nm corresponds to a bandgap of 1.65 eV for the film. The quenching of the photoluminescence of the DPP macroinitiator observed in the polymer was attributed to a photoinduced electron transfer from the donor block to the PBI acceptor. Photovoltaic devices of structure ITO/PEDOT:PSS/**8**/LiF/Al were fabricated and characterized under a AM 1.5 solar simulator at 100 mW cm^−2^. Cells based on films cast from chloroform solution gave a PCE of only 0.03% with *J*_sc_ = 0.19 mA cm^−2^. Replacement of chloroform by chlorobenzene, reduction of the film thickness from 120 to 70 nm, and solvent annealing with dichloromethane led to a large improvement of PCE to 0.89% with *J*_sc_ increasing to 4.30 mA cm^−2^.^79^

**Scheme 4:**
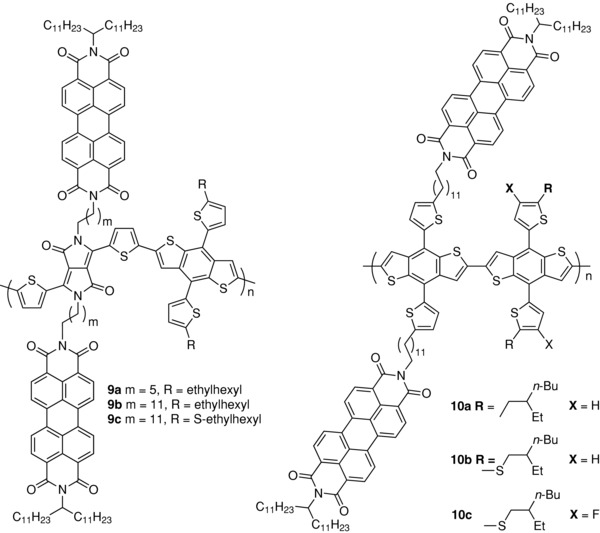
Chemical structure of polymers **9** and **10**.

**Figure 2:**
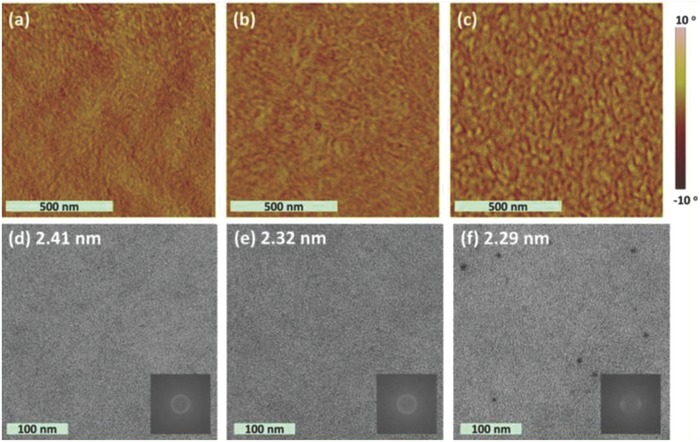
a–c) AFM phase images and d–f) bright‐field TEM images of the polymers thin films fabricated from chlorobenzene diiodooctane solution and annealed at 150 °C for 10 min. (a, d) **10a**, (b, e) **10b**, and (c, f) **10c**. Insets: Fourier transform of part of the images to determine *d*‐spacings. Reproduced with permission.^82^ Copyright 2017, American Chemical Society.

**Figure 3:**
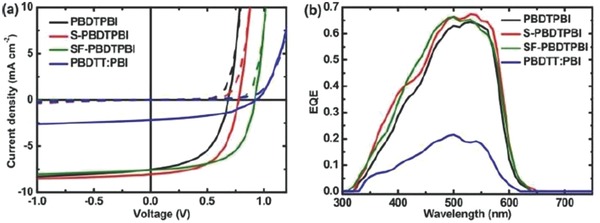
a) *J*–*V* characteristics of solar cells based on polymers **10** (dashed line in the dark and solid line under white light illumination. b) EQE of optimized SMOSCs. PBDTPBI: **10a**, S‐PBDTPBI: **10b**, SF‐PBDTPBI: **10c**. Reproduced with permission.^82^ Copyright 2017, American Chemical Society.

Li and co‐workers have recently described the synthesis of conjugated polymers combining dithienylbenzodithiophene (DTBDT) as the donor block and dithienyl‐DPP bearing PBI units connected by alkyl linkers as the acceptor unit (**9**) (**Scheme** **4**).^80^, ^81^ Three polymers with alkyl spacers of 6 and 12 carbons and DTBDT substituted by branched alkyl and alkylsulfanyl groups were synthesized. The polymers show broad absorption spectra in the 300–900 nm range with two distinct regions corresponding to the pendant PBI (400–600 nm) and the conjugated polymer backbone (600–900 nm). Increasing the length of the alkyl spacer from 6 to 12 carbons between **9a** and **9c** produces a small decrease of the bandgap from 1.45 eV for **9a** to ≈1.40 eV for **9b** and **9c**, while the resolution of the vibrational fine structure of the long wavelength absorption band increases. Such changes are consistent with both an enhanced planarity and rigidity of the π‐conjugated backbone and stronger π‐stacking interactions. Furthermore, changes in the absorption features of the pendant PBI groups are also consistent with a stronger stacking of PBI groups when longer spacers are used. The length of the alkyl spacer also influences the quenching of the photoluminescence of the polymer backbone with a quenching ratio decreasing from 0.37 for **9a** to 0.18 and 0.09 for **9b** and **9c**, respectively, a difference consistent with a more pronounced nanophase separation. SMOSCs of inverted configuration have been fabricated with the three polymers. Polymer **9a** leads to a *J*_sc_ of 3.21 mA cm^−2^ and PCE of 0.51% increasing to 1.64% and 2.74% for **9b** and **9c**, respectively, while the fill factor also increases from 0.29 to 0.46 (Table 1). The analy‐sis of the morphology of the film by grazing incidence wide angle X‐ray scattering (GIWAXS) revealed an increasing crystallinity from **9a** to **9c**. These results confirm that the optimization of the linker is a crucial parameter that controls not only phase separation but also indirectly the packing arrangement of the donor and acceptor subcomponents.^80^ The authors have compared polymer **9c** with a parent systems containing only one PBI unit on the DPP block.^81^ The results confirmed the higher PCE of the doubly substituted polymer **9c** (2.74% vs 1.20% for the singly substituted polymer) in spite of a lower hole mobility for polymer **9c** (μ_H_ = 1.4 × 10^−2^ vs 3.4 × 10^−2^ cm^2^ V^−1^ s^−1^.^81^ More recently, the same group has reported the synthesis of another series of parent “double‐cable” polymers with poly(DTBDT) as supporting backbone and pendant PBI acceptor groups (**10**).^82^ The use of a linear π‐conjugated backbone of poly(DTBDT) was motivated by an expected improved crystallinity and nanophase separation. The pendant PBI groups were connected to the conjugated backbone by flexible dodecyl alkyl linkers while the effects of substitution of the lateral thienyl group of DTBDT were investigated. The polymers were synthesized by Stille coupling between a dibromo‐DTBDT carrying two PBI side groups and three different distannyl‐BDT compounds substituted by 2‐(2‐ethylhexyl)‐thiophene, 2‐((2‐ethylhexyl)thio)‐thiophene, and 2‐((2‐ethylhexyl)thio)‐3‐fluorothiophene.^82^ The polymers show number‐average molecular weights of 42.8, 37.0, and 26.3 kDa for **10a**, **10b**, and **10c**, respectively. The absorption spectra of the polymers show two peaks at 490 and 530 nm in thin films and solution and a shoulder at 560 nm attributed to stacked PBI units since a similar shoulder is observed in the spectrum of PBI. The polymers have an optical bandgap (*E*_g_) of 2.10 eV, similar to that poly(DTBDT) and of PBI units. SMOSCs of inverted configuration ITO/ZnO/**10**/MoO_3_/Ag were fabricated with the three polymers. Active films were spun‐cast from chlorobenzene solutions with diiodooctane as the additive and thermally annealed at 150 °C (**Figure** **2**). The best cell based on **10a** gave a PCE of 2.72% increasing to 3.60% and 4.18% for **10b** and **10c**, respectively. The SMOSCs based on the three polymers exhibited rather comparable *J*_sc_ values of ≈8.0 mA cm^−2^ but presented significant difference in *V*_oc_ which increases from 0.69 V for **10a** to 0.92 for **10c** (Table 1). The high PCE obtained with this type of “double‐cable” polymer corroborated by maximum EQE values > 65% (**Figure** **3**) has been attributed to the optimized nanophase separation between the π‐conjugated backbone and the side PBI groups (Figure 2) and to a better balanced ratio of hole and electron mobilities than for a two‐component reference system.^82^

### 2.3 Block Copolymers with “In‐Chain” Acceptor Groups

The above discussed “double‐cable” polymers in which acceptor groups are connected via flexible linkers onto a main donor polymer backbone has been clearly inspired by the BHJ model. The optimization of the D/A nanophase separation with separated packing and eventual specific crystallization of D and A is expected to favor exciton diffusion and charge separation, to limit charge recombination, and to facilitate the transport of positive and negative charges toward their respective electrodes. A quite different strategy consists in the insertion of both the D and A units in the same linear, eventually fully conjugated, polymer backbone. As for all SMOSC materials in general, one of the major problems is the creation of the optimal nanophase separation of D and A domains. One of the first example of such “in‐chain” D–A polymers was reported in 2009 by Mikroyannidis et al. who synthesized an alternating PPV–PBI copolymer. However, the efficiency of the resulting SMOSC did not exceed 0.02%.^83^

**Scheme 5:**
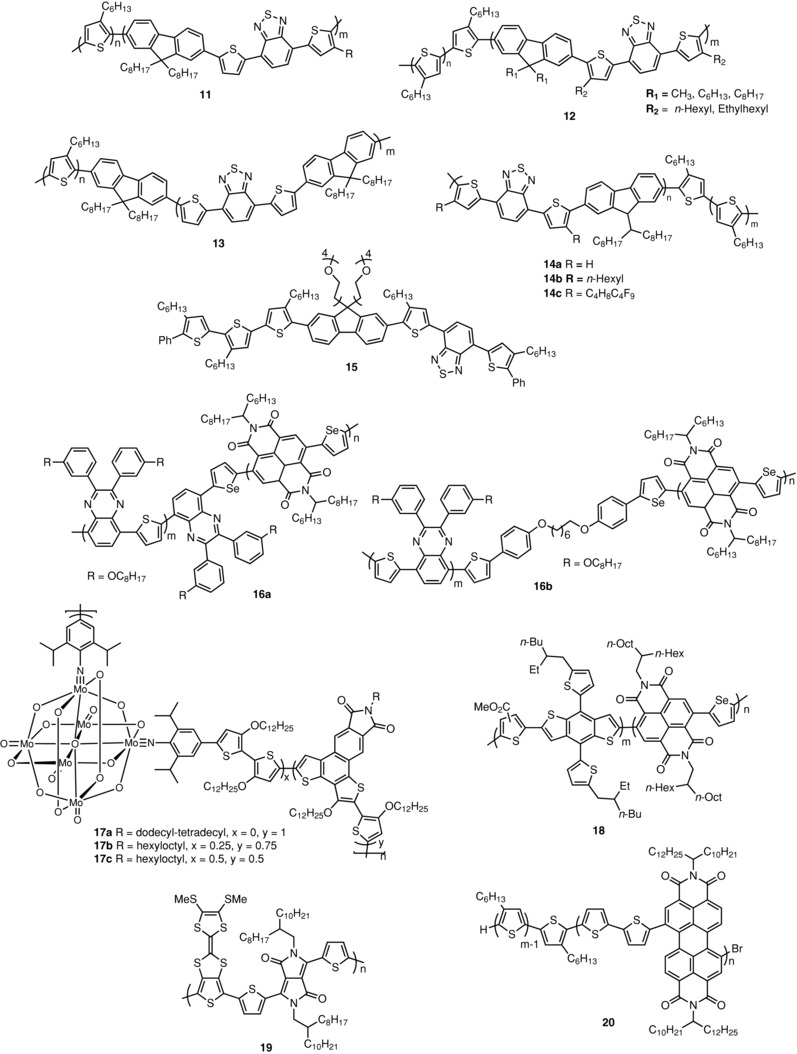
Chemical structure of copolymers **11**–**20**.

Guo et al. synthesized all‐conjugated poly(3‐hexylthiophene)‐*block*‐poly((9,9‐dioctylfluorene)‐2,7‐diyl‐*alt*‐[4,4‐bis(thiophen‐5‐yl)‐2,1,3‐benzothiadiazole]‐2′,2″‐diyl block copolymers (**11**) as active material for SMOSCs (**Scheme** **5**).^84^, ^85^ The copolymers were synthesized by GRIM and Suzuki–Miyaura polycondensation. GRIM is first carried out to give a bromo end‐functionalized P3HT macroreagent which is subsequently submitted to Suzuki–Miyaura polycondensation with various diboronic esters.^84^ The copolymer containing dioctylfluorene (**11**) has a total weight‐averaged MW of 29 kDa with 56% weight P3HT. These copolymers self‐assemble into in‐plane lamellar morphologies with alternating D and A domains with characteristic size of 9 nm (*d*‐spacing of 1 nm). SMOSCs of 16 mm^2^ active area were prepared. It was found that the temperature and duration of thermal annealing have a strong influence on cell performances with PCE increasing from 1.5% for 20 min at 100 °C to 2.7% for 10 min at 165 °C. The best device gave a *J*_sc_ of 5.20 mA cm^−2^ and a PCE of 3.10%, which is the highest value reported for a SMOSC based on a diblock copolymer. All devices present high *V*_oc_ values in the range of 1.10–1.20 V.^84^ The same group has reported the synthesis of a library of copolymers with various substitution patterns on the fluorene and thiophene units (**12**) in order to analyze their effects on the solubility and packing of the polymers.^86^ All polymers present a face‐on orientation with π‐stacking direction in the out‐of‐plane direction. However, it was found that the thermal annealing conditions strongly affect crystal orientation. Despite the large number of new copolymers synthesized, all SMOSCs based on these materials presented PCE inferior to 0.10%, leading to the conclusion that the introduction of solubilizing chains on the thiophene rings has a detrimental effect on phase segregation.^86^ More recently, the same group has investigated the effect of the mode of linkage of the D and A blocks in the same class of block copolymers.^87^ Thus, a new sample of **11** (P3HT–PTBTF) with MW of 20 kDa and a P3HT content of 70 wt% was compared to a parent system in which the P3HT donor block and the dithienyl benzothiadiazole acceptor block were connected by a fluorene unit (**13**). The best cells based on P3HT–PTBTF gave a PCE of only 0.05%, instead of the previously reported 3.0% for a cell based on a sample of **11** of higher MW and lower P3HT content.^87^ Although both copolymers present rather similar absorption spectra, the insertion of the fluorene bridge in the structure was reported to produce a large increase of PCE to 2.24%, a result attributed to more efficient charge separation in **13**.^87^

Lombeck et al. have synthesized conjugated D–A block copolymers with carbazole donor units and dithienyl benzothiadiazole acceptor blocks (**14**) in order to investigate the effects of substitution of the two thienyl rings on phase separation and conversion efficiency.^88^ The semifluorinated octyl‐substituted dithienyl benzothiadiazole block was obtained by direct arylation which was reported as a crucial step for successful synthesis of the block. Partial fluorination of the alkyl chains (**14c**) leads to an almost full recrystallization of the P3HT block in the copolymers. In contrast, crystallization is drastically reduced with hydrogen and hexyl substituents (**14a** and **14b**) due to a partial miscibility between the two segments. No phase separation was observed for **14a** and **14b** while for **14c,** a phase‐separated structure with a spacing of 9.5 nm was obtained. Furthermore, for **14c**, a face‐on orientation of P3HT was observed in contrast to **14a** and **14b**. SMOSCs of structure ITO/PEDOT:PSS/**14**/aluminum were fabricated. Polymer **14a** with unsubstituted thiophenes leads to inefficient devices. The introduction of hexyl chain (**14b**) improves all parameters *J*_sc_, *V*_oc_, and FF, leading to a PCE of ≈0.50%, whereas replacement of the hexyl chains by semifluorinated octyl chains (**14c**) leads to a twofold increase of PCE to 0.95%.^88^

Mitchell et al have synthesized a block copolymer based on the same D and A units but with tetraethyleneglycol chains on the fluorene unit (**15**) in order to amplify phase separation in the material. This type of substitution was reported to facilitate the purification of the polymer and to lead to better defined morphologies. However, the polymer presented very low electron mobility and led to poorly efficient solar cells.^89^

While many block copolymers for OPV contain a large fraction of P3HT, Lee et al. have synthesized one of the few examples of SMOSC based on copolymers containing less common building blocks. Two types copolymers composed of quinoxaline–thiophene and naphthalene dicarboximide–selenophene blocks (**16**), in which the two blocks are connected either by direct conjugation (**16a**) or through a flexible 6‐carbon alkyl spacer (**16b**), were synthesized.^90^ The polymers present *M*_n_ of 11.9 and 12.4 kDa and PDI of 2.22 and 2.12 for **16a** and **16b**, respectively.^90^ Gas phase chromatography (GPC) analyses indicated that both the polymers contain a 1:1 ratio of donor and acceptor blocks. OPV cells of inverted architecture ITO/ZnO/**16**/MoO_3_/Al were fabricated with **16a**, **16b**, and a blend of the two homopolymers. Cells based on this blend gave a *J*_sc_ = 1.61 mA cm^−2^, *V*_oc_ = 0.67 V, and FF = 0.37, leading to a PCE of 0.40%. A SMOSC based on polymer **16a** led to a slightly lower efficiency with *J*_sc_ = 1.30 mA cm^−2^, FF = 0.35, and PCE = 0.36%. Finally, the insertion of a flexible spacer between the D and A blocks in **16b** induces a net increase of performances with *J*_sc_ = 4.04 mA cm^−2^ and PCE = 1.54%. This large improvement, due in particular to a better FF, and confirmed by the EQE spectra, was attributed to a limitation of charge recombination by the spacer in **16b**.^90^

Taking advantage of the specific size, geometry, and electron acceptor properties of polyoxometalates, organic–inorganic copolymers containing various amounts of hexamolybdate clusters have been synthesized (**17**).^91^ Hexamolybdate clusters combine a spherical shape of dimensions close to those of C_60_ (diameter 0.8 vs 0.7 nm for C_60_) and a reduction potential close to that of C_60_. Conjugated block copolymers were synthesized by coupling of a bithiophenic Stille reagent with 2,5‐dibromo‐dithienobenzoisoindole‐dione and diiodo‐functionalized hexamolybdate. A reference polymer devoid of hexamolybdate cluster (**17a**) was reported to give a PCE of 2.45% in BHJ cells with PC_71_BM as the acceptor and 3% of diiodooctane as the additive. Two copolymers with contents of hexamolybdate cluster close to the feed ratio (*x* and *y*) were synthesized. The copolymers presented averaged MW of 5.48 and 3.69 kDa and start to decompose at 200 °C. Thermal stability seems to decrease with the increase of the cluster content. Both the copolymers show blueshifted absorption maxima with reference to polymer **17a** (509 and 515 nm for **17b** and **17c**, respectively, vs 535 nm for **17a**), suggesting that the cluster produces some steric obstacles to the planarity of the π‐conjugated backbone. SMOSCs of 14 mm^2^ active areaof structure ITO/PEDOT:PSS/**17**/Ca/Al were fabricated and characterized under AM 1.5 simulated solar light. Devices based on the reference polymer **17a** gave a *J*_sc_ of 60 µA cm^−2^ and low PCE of ≈0.01%. The best results were obtained with copolymer **17b** with the lowest cluster content (*x* = 0.25). The cell gave a maximum PCE of 0.31% with a *J*_sc_ of 3.25 mA cm^−2^. The cells exhibit rather low open‐circuit voltage, while the very low FF values could reflect some problems of charge transport.^91^

Very recently, Choi, and co‐workers reported another example of conjugated non‐P3HT‐based copolymer. In this work, a wide bandgap donor block of DTBDT–thiophenecarboxylate is connected to a narrow bandgap naphthalenediimide–selenophene acceptor block in order to obtain a wide complementary absorption (**18**).^92^ The copolymer was synthesized by Stille coupling of oligomeric blocks of the D and A components. The crude product was purified by Soxhlet extraction with solvents of various polarities. The D and A oligomeric blocks and the D–A copolymer presented *M*_n_ of 6.84, 10.7, and 18.1 kDa, respectively, with PDIs of 1.53, 2.16, and 2.31. The UV–vis absorption spectrum of polymer **18** in solution exhibits the characteristic features of the D and A blocks. The spectrum of solution‐cast film shows a maximum at 528 nm and a bandgap of 1.80 eV. The photovoltaic properties of the polymers were analyzed in SMOSCs of inverted architecture: ITO/ZnO/**18**/MoO_3_/Ag, using a cell based on a mixture of the D and A oligomeric materials as reference. This latter device shows a PCE of 1.14% with a *J*_sc_ of 3.05 mA cm^−2^, a *V*_oc_ of 0.86 V, and a FF of 0.43. However, the SMOSC fabricated with **18** delivered a *J*_sc_ of 8.30 mA cm^−2^, a *V*_oc_ of 0.93 V, and a FF of 0.50, leading to a PCE close to 4.0% which ranks among the best values reported for a polymer‐based SMOSC and the highest for an “in‐chain” system.^92^

**Figure 4:**
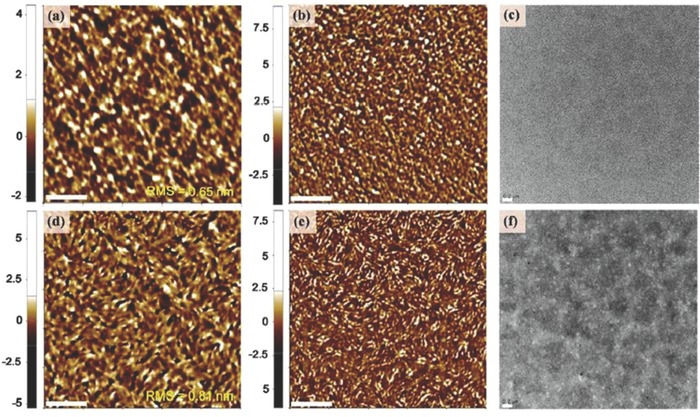
a,b,d,e) AFM images (scale bar = 1 µm) and c,f) TEM images (scale bar = 200 nm) of the active layer in OPV devices. (a) **18**, (d) blend film. Phase images of films fabricated with (b) **18**, (e) blend film. TEM images: (c) **18**, (f) blend film. Reproduced with permission.^92^ Copyright 2018, American Chemical Society.

As shown in **Figure** **4**, AFM images of the as‐cast films of the blend of the oligomeric blocks exhibit larger surface domains than films of copolymer **18** which display a uniform, smooth morphology. Furthermore, TEM shows that films of **18** present a well‐defined nanophase segregation and a much more uniform internal morphology than the blend films.

Skabara and co‐workers have synthesized a D–A polymer combining a dithienyl‐DPP electron acceptor group and a fused thieno‐tetrathiafulvalene (TTTF) moiety as electron donor (**19**) (Scheme 5). The introduction of the strong TTF donor in the structure was expected to improve hole transport.^93^, ^94^ The polymer was synthesized by Suzuki–Miyaura cross‐coupling of a dibromo‐TTTF and the diboronic ester of a dithienyl‐DPP. The UV–vis absorption spectrum of polymer films shows a broadband in the 600–1000 nm region and hence an adequate bandgap for harvesting visible light. Organic field‐effect transistors (OFETs) based on films of the polymer spun‐cast from solutions in different solvents gave hole mobilities of 2–7 × 10^−2^ cm^2^ V^−1^ s^−1^. BHJ cells were fabricated using PC_71_BM as the acceptor in a 1:4 ratio. A BHJ cell cast from dichlorobenzene solutions of **19** and PC_71_BM gives a PCE of 1.80% with *J*_sc_ of 8.0 mA cm^−2^, a *V*_oc_ of 0.71 V, and a FF = 0.32.^93^ On the other hand, a SMOSC of structure ITO/PEDOT:PSS/**19**/Ca/ Al exhibited a *J*_sc_ of 1.80 mA cm^−2^, a *V*_oc_ of 0.61 V, and a PCE of 0.30%.^93^

Wang et al. have synthesized block copolymers combining P3HT donor and PBI (**20**).^95^ The copolymers were purified by preparative GPC in order to remove the excess of P3HT and to reduce polydispersity. The copolymers were divided into several fractions and it was found that the optical and electrochemical properties strongly depend on the ratio of the P3HT and PBI blocks. SMOSCs of structure ITO/PEDOT:PSS/**20**/Ca/Al were fabricated and characterized under AM 1.5 simulated solar light. The best results were obtained with a copolymer of the lowest PDI (1.23) containing 43% of P3HT. A PCE = 0.06% was obtained with the as‐cast film, increasing to 1.00% after annealing at 150 °C.^95^ Under the same conditions, a SMOSC ITO/PEDOT:PSS/**20**/Al gave after 20 min thermal treatment at 120 °C a *J*_sc_ of 1.80 mA cm^−2^, a *V*_oc_ of 0.61 V, and a PCE of 0.30%. This modest value was attributed to the inhomogeneity of the film and to unbalanced charge transport due to the low electron mobility of the polymer.^95^

To summarize, a large part of the work on SMOSCs has been devoted to “double‐cable” polymers with pendant fullerene acceptor groups. Developed in the frame of the BHJ paradigm, this approach relies for a large part on the optimization of the linker connecting the A unit to the π‐conjugated backbone in order to control nanophase separation and specific self‐organization with eventual crystallization of D and/or A. Major progress has been recently accomplished leading in some cases, to PCE comparable to the best results obtained with multicomponent cells. Some first examples of highly efficient “double‐cable” polymers with NFA have been described recently and the rapid expansion of NFA will probably lead to significant progress in a near future. Much effort has been invested in the synthesis of block copolymers with controlled nanophase separation but progress in this direction is slow. These materials are often difficult to purify and their properties are very sensitive to their processing conditions. Thus, the high PCE of 3.0% published some years ago has not been matched yet. On the other hand, PCE close to 4.0% has been recently obtained with fully conjugated D–A polymers. These promising results show that besides “double‐cable” polymers, “in‐chain” D–A systems have also a great potential for the design of efficient SMOSC materials.

## 3 SMOSCs Based on Molecular Active Materials

The advantages of molecular donors versus polymers as active OPV materials have been extensively discussed in particular regarding the reproducibility of synthesis and purification and the analysis of structure–properties relationships.^30^, ^31^, ^32^, ^33^, ^34^, ^35^, ^36^, ^37^, ^38^, ^40^, ^96^ In the specific case of SMOSCs, it seems clear that at such an early stage of research, molecular systems can significantly contribute to better understand the relevant structural factors and thus to progress toward the definition of synthetic principles for the design of active materials. A huge number of dyads combining fullerenes and molecular π‐conjugated systems have been synthesized.^16^, ^17^, ^18^, ^19^, ^60^, ^61^, ^62^, ^63^ In most cases, these compounds were synthesized as models of molecular heterojunctions for the analysis of the elemental mechanisms of photoinduced electron and/or energy transfer and only a few of them have been evaluated in SMOSCs. As for polymer‐based materials, molecular materials for SMOSCs can be also divided into “side‐chain” and “in‐chain” systems.

### 3.1 Fullerene‐Based Nonconjugated Molecular D–A Systems

**Scheme 6:**
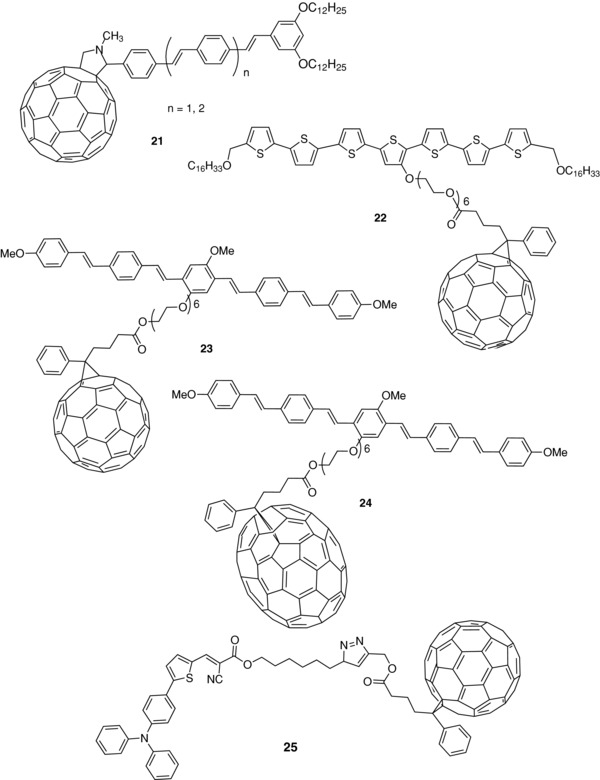
Chemical structure of dyads **21**–**25**.

One of the first examples of molecular dyad specifically designed for photovoltaic conversion was reported in 1999 by Nierengarten et al.^97^, ^98^ Several dyads consisting of phenylenevinylene oligomers (*n*PVs) covalently fixed on fullerene C_60_ through a pyrrolidine group were synthesized and evaluated in SMOSCs. The best results were obtained with compound **21** (**Scheme** **6**) which gave current of ≈10 µA under monochromatic irradiation.^98^

Hashimoto and co‐workers have carried out extensive work on the synthesis of active materials for SMOSCs based on both polymeric and molecular structures.^70^, ^71^, ^72^, ^73^, ^99^, ^100^, ^101^ A first example published in 2007 consisted of a conjugated thiophene heptamer with a C_60_ unit attached at the median thiophene ring via an oligo(oxyethylene) chain (**22**).^99^ The presence of two long terminal alkyl chains on the molecule leads to solution‐cast films containing long supramolecular fibrous nanostructures. Compound **22** absorbs at 440 nm in chloroform solution and at 420 nm as cast film. This blueshift was attributed to the formation of H‐aggregates of oligothiophenes in the solid state.

**Figure 5:**
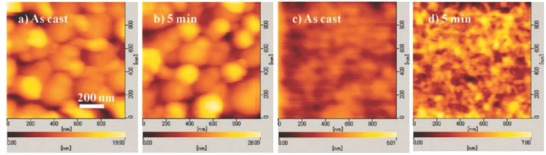
AFM images of a,b) mixture blend of *n*PV:PC_71_BM; c,d) **24**. (a) and (c): as‐cast films, and (b) and (d) after 5 min at 110 °C. Reproduced from ref. 92 with permission from PCCP Owner Societies.

Under standard AM 1.5 conditions, a SMOSC ITO/PEDOT:PSS/**22**/Al gave a *J*_sc_ of 0.93 mA cm^−2^ and a PCE of 0.15% (**Table** **2**).^99^ Two years later, the same group reported the synthesis of an *n*PV containing five phenyl rings with a C_60_ unit attached to the middle of the chain by a polyether linker (**23**).^100^ X‐ray diffraction and thermal data of solution‐cast films suggested crystallinity associated with the π‐stacking of *n*PV chains. The UV–vis absorption spectrum shows a λ_max_ at 423 nm in solution (assigned to the *n*PV chain), shifting to 439 nm for the film, a redshift attributed to the formation of J‐aggregates. A device ITO/PEDOT:PSS/**23**/polydimethylsiloxane‐*b*‐methylmetacrylate/Al gives a *J*_sc_ of 3.30 mA cm^−2^ and a PCE of 1.28%.^100^ Although the ≈1 order of magnitude increase of PCE compared to compound **22** confirms the importance of the mode of molecular packing and crystallinity, a direct comparison is difficult due to the presence of an additional buffer layer in the cell based on **23**. More recently, the same group reported the synthesis of a parent compound involving the same *n*PV platform and linker but with a C_70_ unit instead of C_60_ (**24**).^101^ As in the case of **23**, the UV–vis absorption spectrum presents a maximum at 440 nm with an additional shoulder at 480 nm, leading to an extension of the absorption in the visible range due to the presence of C_70_. The efficiency of the dyad was evaluated on a device of structure ITO/PEDOT:PSS/**24**/PC_61_BM/Ca/Al. Before deposition of PC_61_BM, calcium, and aluminum, the devices were thermally annealed 5 min at 110 °C under nitrogen. As shown by AFM images (**Figure** **5**), films of dyad solution exhibit a smaller surface roughness interpreted as a reduction of large phase separation. The EQE spectrum of the complete device shows a maximum of 45% at 480 nm. When irradiated in AM 1.5 conditions, the cell give a *J*_sc_ of 4.60 mA cm^−2^ (instead of 3.30 mA cm^−2^ for the C_60_‐based device), leading to a PCE of 1.92%. Despite an obvious improvement of performances due to the replacement of C_60_ by C_70_, a strict comparison with previous results is again difficult due to the presence of additional layers of PC_61_BM and calcium.

**Table 2:** Cell structure and photovoltaic characteristics of SMOSCs based on molecular photoactive materials under simulated AM 1.5 solar light. PL: photoactive layer; aa: active area; SA: standard anode ITO/PEDOT:PSS; PDMS: poly(dimethylsiloxane)

Compound	Cell structure	aa [mm^2^]	EQE_max_	*J_s_*_c_ [mA cm^−2^]	*V*_oc_ [V]	FF	PCE [%]	Ref.
**22**	SA/PL/Al	–	0.31	0.93	0.70	23	0.15	99
**23**	SA/PL/ PDMS/Al	–	–	3.30	0.88	44	1.28	100
**24**	SA/PL/PC_61_BM/Ca/Al	12	0.45	4.60	0.91	46	1.92	101
**25**	SA/PL/Ca/Al	10	0.28	2.10	0.73	29	0.40	102
**26a**	SA/PL/Ca/Al	6	0.07	0.69	0.60	25	0.10	103
**26b**	SA/PL/Ca/Al	6	0.16	3.17	0.56	33	0.56	103
**26c**	SA/PL/Ca/Al	6	0.28	4.79	0.50	46	1.11	103
**26d**	SA/PL/Al	6	0.17	3.80	0.59	32	0.72	104
**27**	SA/PL/Al	6	0.21	4.50	0.57	33	0.84	104
**28**	SA/PL/Al	–	0.05	0.31	0.91	28	0.08	105
**29a**	SA/PL/Al	3	–	2.67	0.66	28	0.49	106
**29b**	SA/PL/Al	3	–	1.43	0.73	26	0.27	106
**29c**	SA/PL/Al	3	–	1.30	0.71	25	0.23	106
**30a**	SA/PL/LiF/Al	4	0.37	2.64	0.51	33	0.43	109
**30b**	SA/PL/LiF/Al	4	0.28	1.50	0.42	–	0.19	109
**31**	SA/PL/Ca/Al	–	0.30	1.75	0.79	27	0.40	110
**32**	SA/PL/ZnO/Al	–	0.40	7.02	0.97	36	2.40	111
**34**	ITO/MoOx/PL/Ca/Al	–	0.38	6.71	0.66	49	2.17	116
**35a**	SA/PL/HBL/LiF/Al	3.14	–	3.16	0.90	25	0.70	118
**35b**	SA/PL/HBL/LiF/Al	3.14	–	4.05	0.86	34	1.18	118
**35c**	SA/PL/HBL/LiF/Al	3.14	0.46	4.49	0.87	38	1.50	118
**36a**	SA/PL/HBL/LiF/Al	14	0.25	2.33	0.84	22	0.42	120
**36b**	SA/PL/HBL/LiF/Al	14	0.48	4.92	0.85	32	1.32	120
**36c**	SA/PL/HBL/LiF/Al	14	0.60	5.90	0.88	35	1.75	120
**37a**	SA/PL/HBL/LiF/Al	–	0.58	4.30	1.00	43	1.86	121
**37b**	SA/PL/HBL/LiF/Al	–	0.64	4.82	1.04	47	2.33	121
**37c**	SA/PL/HBL/LiF/Al	–	0.61	4.60	0.96	46	2.04	121
**38**	SA/PL/HBL/LiF/Al	–	0.62	5.32	1.04	49	2.70	121
**39a**	SA/PL/Al	–	–	1.55	0.55	0.45	0.38	123
**39b**	SA/PL/Al	–	–	1.22	0.38	0.38	0.35	123
**39c**	SA/PL/Al	–	–	2.36	0.51	0.45	0.54	123
**40a**	SA/PL/Al	–	–	1.05	0.37	0.37	0.12	123
**40b**	SA/PL/Al	–	–	0.98	0.40	0.40	0.15	123
**40c**	SA/PL/Al	–	–	1.52	0.49	0.40	0.30	123
**41**	SA/PL/Al	28	–	1.70	0.76	0.27	0.40	31

The thermal stability of the cell based on **24** has been compared to that of two‐component BHJ cells fabricated by mixing the same *n*PV bearing a polyether linker with a terminal hydroxyl group with PC_71_BM. AFM images of both the types of films subjected to various annealing conditions revealed that films of the dyad have smaller surface roughness, suggesting a reduced phase separation (Figure 5). The time‐dependent stability under thermal annealing of the photovoltaic parameters *J*_sc_, FF, *V*_oc_, and PCE was investigated for BHJ and SMOSC cells. After 3 days of thermal annealing at 110 °C, the BHJ cell shows an almost threefold decrease of PCE from 0.65% to 0.23%. Under the same conditions, the cell based on the dyad still exhibits a PCE as high as 1.52%. This remarkable result clearly confirms the interest of SMOSCs for the production of stable solution‐processed OPV cells.^101^

Labrunie et al. have synthesized a molecular dyad involving a triphenylamine‐based push–pull system and C_60_ (**25**) by Huisgen‐type click chemistry.^102^ A significant quenching of the photoluminescence of the push–pull moiety was observed in solution and a complete quenching in line with ultrafast photoinduced electron transfer was observed on solution‐processed film. Under AM 1.5 simulated solar light, a SMOSC PEDOT:PSS/**25**/Ca/Al presented a *V*_oc_ of 0.73 V, a *J*_sc_ of 2.10 mA cm^−2^, and a FF of 0.29, leading to a PCE of 0.40%. Evaluation of the charge carrier mobility by the space‐charge limited current method gives an electron mobility (*µ*_e_) of 4.3 × 10^−4^ cm V^−1^ s^−1^, ≈50 times higher than the hole mobility.^102^ A significant level of charge recombination was attributed to these unbalanced charge mobilities.

**Scheme 7:**
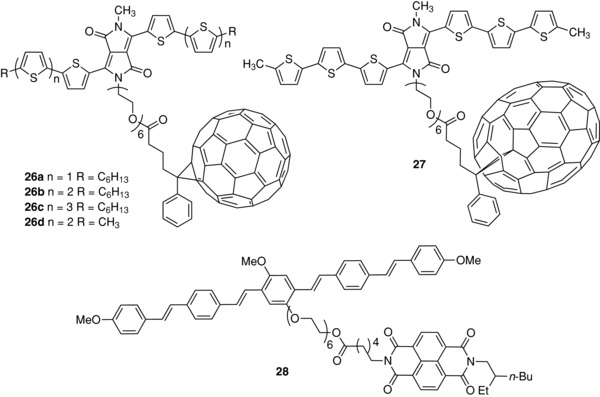
Chemical structure of dyads **26**–**28**.

Tajima and co‐workers have synthesized a series of dyads in which C_60_ is connected to a hybrid tricyclic system consisting of a DPP unit flanked by two short‐chain oligothiophenes (**26**) (**Scheme** **7**).^103^ The UV–vis absorption spectrum of these compounds is dominated by a broadband in the 500–700 nm region. As expected, increasing the number of thiophene rings leads to a bathochromic shift of the absorption edge. The presence of the attached fullerene produces a quenching of fluorescence emission indicative of a charge transfer between the conjugated system and C_60_. The same group has reported a bis‐terthienyl‐DPP donor system (3T‐DPP‐3T) with a grafted C_70_ unit (**27**).^104^ Comparison with the absorption spectrum of films of 3T‐DPP‐3T devoid of fullerene but with methyl and hexyl terminal groups suggests an absence of interaction between the donor and C_60_ in **27** in the ground state. On the other hand, the larger redshift of the absorption edge of the methyl compound and the exaltation of the vibrational fine structure are consistent with a stronger π‐stacking, in agreement with X‐ray diffraction results. More recently, the same group has synthesized another *n*PV‐based dyad in which fullerenes were replaced by naphtalenediimide (NDI) (**28**).^105^ The absorption spectrum was the simple sum of the spectra of NDI and conjugated chain with a maximum at 420 nm and additional peaks at 342, 360, and 380 nm due to the NDI moiety. Fluorescence emission spectra revealed that the attached NDI produces a significant quenching of the emission intensity of the *n*PV chain, consistent with an intramolecular electron transfer to NDI. However, the quenching of fluorescence was less intense than in the case of the dyad with attached C_70_. X‐ray diffraction measurements of the films showed that contrary to the parent fullerene‐based dyads, *n*PV and NDI aggregate and crystallize separately in the solution‐cast films, which represents an important result for the control of the nanoscale order in dyad‐based SMOSCs.^105^

**Scheme 8:**
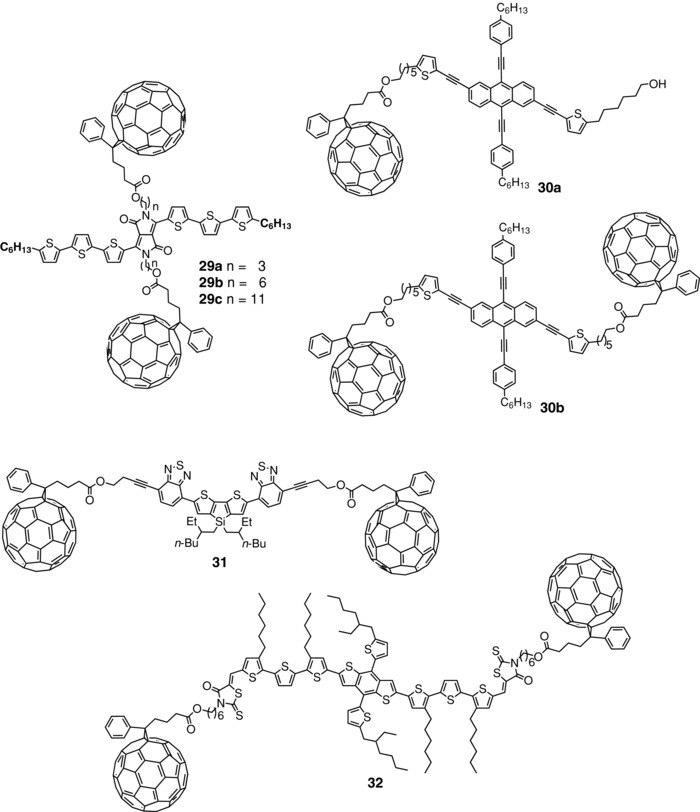
Chemical structure of dyads and triads **29**–**32**.

Whereas in dyads **21**–**28**, only one C_60_ block is attached to the conjugated donor chain, Chen et al. synthesized a 3T‐DPP‐3T donor with two C_60_ units attached at both nitrogen atoms of DPP via alkyl linkers of 3, 6, and 11 carbons (**29a**–**c**) (**Scheme** **8**).^106^ The absorption spectra show a broadband extending from 250 to 800 nm with a maximum at 630 nm due to the internal charge transfer in the 3T‐DPP‐3T system. The potential of the three compounds was evaluated in SMOSCs of structure ITO/PEDOT:PSS/**29**/Al. As shown in Table 2, all compounds lead to modest performances with *J*_sc_ and PCE values that decrease with the lengthening of the alkyl spacer from 2.67 mA cm^−2^ and PCE = 0.49% for a 3‐carbon spacer (**29a**) to 1.30 mA cm^−2^ and a PCE of 0.23% for the 11‐carbon spacer (**29c**).^106^ The electron mobility of the three compounds determined in OFETs gave much higher value (1.5 × 10^−3^ cm^2^ V^−1^ s^−1^) for **29a** with the shortest linker than for **29b** and **29c** (6.1 × 10^−4^ and 4.5 × 10^−4^ cm^2^ V^−1^ s^−1^), respectively. These values are lower than the electron mobility of PC_61_BM (4.1 × 10^−2^ cm^2^ V^−1^ s^−1^). On the other hand, it was reported that hole mobilities were too low to be detected in OFET devices. In fact DPP‐based donor often presents low hole mobilities, while the PCE of this class of donor is extremely sensitive to small structural changes.^107^, ^108^ The authors have attributed the low PCE to the presence of the insulating alkyl spacers and to a decrease of the efficiency of charge‐separation due to the long distance between the donor and acceptor blocks. Such explanations are not fully convincing since several examples of highly efficient SMOSCs have been fabricated with “double‐cable” polymer in which the D and A blocks are connected by long alkyl linkers (see above). On the other hand, the results obtained with conventional BHJ cells based on blends of compounds **29** and PC_61_BM confirm that these molecular donors lead to PCE significantly inferior to those of P3HT.^106^

Chin et al. have described donor–acceptor molecules in which PC_61_BM acceptor units are attached on an anthracene donor block substituted by hexylphenylethynyl groups (**30**).^109^ Two compounds containing one and two C_60_ units were synthesized using Sonogashira coupling. Both the compounds absorb essentially below 530 nm with a bandgap of ≈2.25 eV. SMOSCs of structure ITO/PEDOT:PSS/**30**/LiF/Al were fabricated with spun‐cast films of the two molecules. The singly substituted system **30a** gave the best results with PCE = 0.43% and *J*_sc_ = 2.64 mA cm^−2^, whereas introduction of a second C_60_ in **30b** leads to approximately twofold decrease of the current intensity and efficiency (Table 2).^109^ Cao et al. have reported a dumbbell‐like A–D–A molecule with a dithienosilole‐dibenzothiadiazole donor unit with two fullerene C_60_ side groups (**31**).^110^ The UV–vis spectrum of the donor system presents the typical absorption of C_60_ together with a broad absorption band in the 450–650 region due to the conjugated donor chain. A cell ITO/PEDOT:PSS/**31**/Ca/Al was evaluated under AM 1.5 simulated solar light, giving a PCE of 0.40% with a *J*_sc_ of 1.75 mA cm^−2^. The material presented a hole mobility of 3.6 × 10^−5^ cm^2^ v^−1^ s^−1^ and an electron mobility of 5.7 × 10^−4^ cm^2^ V^−1^ s^−1^. The low FF value of 0.27 was attributed to this unbalanced charge transport.^110^

**Figure 6:**
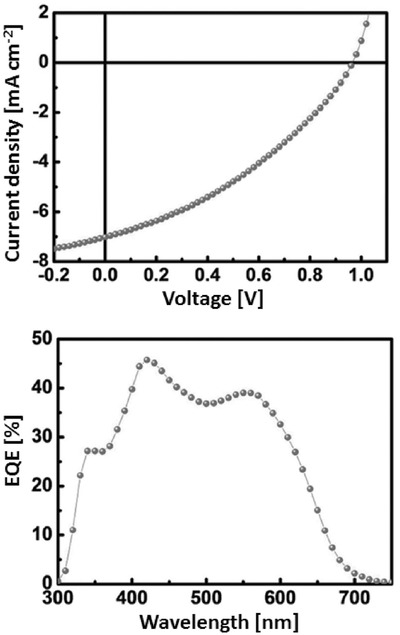
*J*–*V* characteristics (top) and EQE spectrum (bottom) of a SMOSC based on **32**. Reproduced with permission.^111^ Copyright 2018, American Chemical Society.

Very recently, Nguyen et al. have reported the synthesis of a different type of dumbbell molecule consisting of a conjugated block with a central DTBDT unit with two dioctyl terthienyl side blocks and two rodanine terminal acceptors moieties on which two PC_61_BM groups were linked by 6‐carbon alkyl spacers (**32**).^111^ This type of hybrid oligomeric conjugated structure had been previously used as molecular donor in highly efficient bicomponent BHJs.^112^, ^113^ The UV–vis spectrum of films of compound **32** presents a broad absorption band in the 300–700 nm region with a maximum at 557 nm in solution which represents a 20 nm hypsochromic shift compared to the donor system with two terminal hydroxyalkyl chains devoid of C_60_. This blueshift and the attenuation of the 650 nm aggregation shoulder visible in the spectrum of the fullerene‐free molecule may reflect some hindrance to tight π‐stacking of the conjugated donor chains due to the steric effects of the terminal C_60_ groups. A quenching of the photoluminescence emission was observed for solutions of **32**, attributed to intramolecular charge transfer. SMOSCs of structure ITO/PEDOT:PSS/**32**/ZnO/Al were fabricated and evaluated under simulated solar light. Changes in processing conditions or use of additives had no effect on the morphology and electrical properties of the film, indicating that the chemical structure exerts a predominant influence on morphology. The best devices give a high *J*_sc_ > 7.0 mA cm^−2^ and a PCE of 2.31%, in good agreement with the EQE response extending from 300 to 650 nm (**Figure** **6**). These results rank among the highest values reported for a SMOSC based on a molecular material.^111^

**Figure 7:**
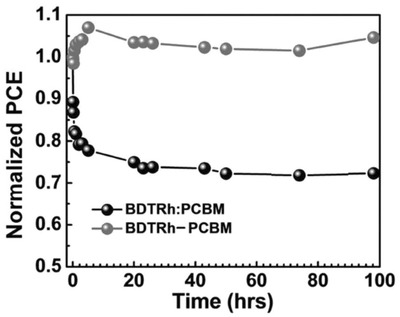
Device stability of SMOSC based on **32** (red) and a blend **32b**:PCBM (black) at an annealing temperature of 80 °C for 100 h. Reproduced with permission.^111^ Copyright 2018, American Chemical Society.

Stability tests performed at 80 °C under inert atmosphere showed that a reference cell based on a blend of PC_61_BM with a donor analog to **32** but devoid of fullerene (**32b**) shows a decay of PCE to 80% of the initial value after 10 min, associated with large phase segregation, suggesting poor morphological stability. In contrast, the SMOSC shows excellent morphological stabilities, with no degradation of PCE after 100 h at 80 °C (**Figure** **7**).

### 3.2 SMOSCs Based on Conjugated Molecular D–A Systems

**Scheme 9:**
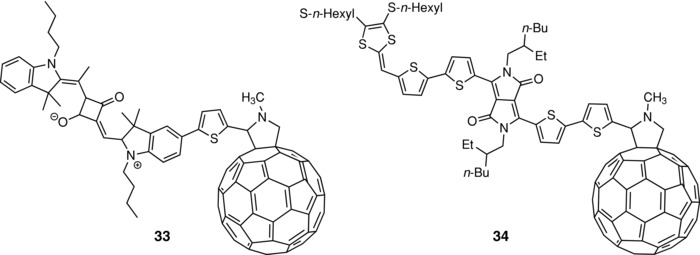
Chemical structures of dyads **33** and **34**.

In many cases, dyads with short in‐chain or through‐space D–A distances suffer from fast geminate recombination of photo‐generated charges.^114^ This problem can be circumvented by the insertion of insulating linkers between the donor and the fullerene unit, according to the BHJ model. Nevertheless, the definition of the optimal structural conditions (mode of connection and distance of D and A units) as well as the minimal spatial extension of D and A blocks needed for charge separation and charge transport remains key problems. In this regard, some recent works indicate that within certain conditions, dyads with a direct fixation of a linearly conjugated chain on C_60_ can lead to materials with interesting photovoltaic properties. Menna and co‐workers have reported a fulleropyrrolidine–squaraine dyad (**33**) (**Scheme** **9**) and investigated its potentialities for the realization of SMOSCs for photodetection. Thus, a SMOSC based on **33** presents an EQE of 0.10 at 700 nm.^115^ Narayanaswamy et al. have synthesized a dyad composed of a dithiafulvalene‐functionalized bis(dithienyl)‐DPP‐fullerene as active material for SMOSCs (**34**).^116^ The introduction of a dithiafulvenyl group in the structure was motivated by an expected increase of donor effect and improved charge transport properties. Films of compound **34** present a broad absorption band in the 500–800 nm region essentially dominated by the absorption of the bis‐dithienyl‐DPP with a maximum at 650 nm. Cells of structure ITO/MoOx/**34**/Ca/Al were tested under monochromatic and white light irradiation. The EQE response extends from 400 to 800 nm with a maximum of 0.38 at 670 nm. Under simulated solar light, the cell gives a *J*_sc_ of 6.70 mA cm^−2^ and a PCE of 2.17% which ranks among the highest values reported for molecular dyads and the best PCE obtained with a dyad with direct connection of C_60_ on the donor.^116^ It is noteworthy that the FF value of 0.49 is much higher than for most of SMOSCs based on dyads reported so far, which suggests that the material presents good hole‐transport properties. This result, which is in striking contrast with initial work on SMOSCs based on C_60_‐based dyads, suggests that the introduction of an electron withdrawing group in the structure of the donor unit contributes to separate the charges and to limit their recombination due to the increase of the dipole moment of the donor block.^117^

As for polymer‐based active materials, two different approaches have been developed for molecular SMOSC materials. The most widely developed is still based on the BHJ paradigm and involves independent D and A moieties connected by an insulating linker in order that D and A self‐assemble into segregated domains of size and continuity, appropriate for exciton diffusion and charge transport. An alternative, less intuitive approach is based on the association of D and A units within the same linear system and with a variable degree of conjugation in order to create a molecular heterojunction.

**Scheme 10:**
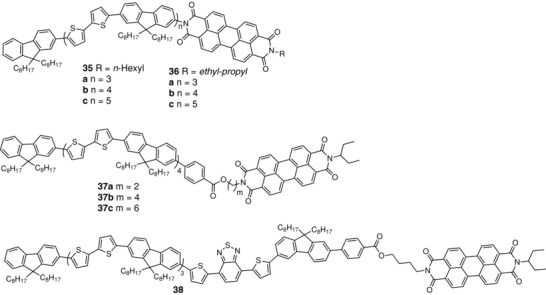
Chemical structures of hybrid oligomers **35**–**38**.

**Figure 8:**
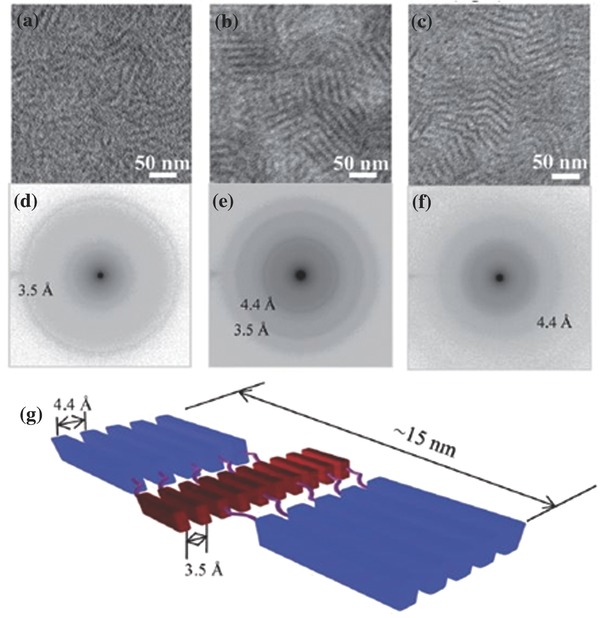
a–c) TEM images and d–f) SAED patterns of **37a**, **37b**, and **37c** thin films at room temperature. g) Schematic illustration of the lamellar nanostructure in the film. Reproduced with permission.^121^ Copyright 2014, Royal Society of Chemistry.

In 2009, Bu et al. reported the synthesis of several extended D–A co‐oligomers combining substituted fluorene‐*alt*‐bithiophene as the donor block and PBI as the acceptor (**35**) (**Scheme** **10**).^118^ These oligomers are smectic liquid crystals. TEM of films showed that the materials are self‐organized into alternating D–A lamellae and it was reported that solvent annealing significantly improved molecular order. Cyclic voltammetry and UV–vis absorption spectra show that the three co‐oligomers have identical oxidation and reduction potentials and similar absorption spectra and bandgaps with the long wavelength region of the spectrum dominated by PBI absorption. This indicates that the effective conjugation length (ECL) is shorter than the geometrical length and that the chain‐length dependence of the convergence limit of ECL is already reached with the shortest molecule.^119^ These similar electronic properties suggest that the role of the large spatial extension of these molecules is essentially to control the self‐organization of the material. The films absorb in the 350–600 nm region and it was proposed that PBI form H‐aggregates. Devices ITO/PEDOT:PSS/**35**/HBL/LiF/Al, where the hole blocking layer (HBL) is *N*,*N′*‐bis(1‐ethylpropyl) perylenediimide were fabricated. The PCE was strongly improved by thermal or solvent annealing processes. The longest system **35c** led to the best results and a SMOSC gave PCE of ≈0.50% for the pristine material increasing to 0.70% after thermal treatment and to 1.50%, with a *J*_sc_ of 4.49 mA cm^−2^, after solvent annealing and insertion of a HBL in the device. The same group has also synthesized another series of the same co‐oligomers in which the hexyl chain attached at the nitrogen atom of PBI was replaced by a branched neopentyl group (**36**). This modification produced some improvement of PCE to 1.75%.^120^ More recently, they have extended their approach to the synthesis of related systems in which the terminal PBI was attached to the hybrid conjugated chain by alkyl linkers containing 2, 4, and 6 carbons (**37**).^121^ The UV–vis absorption spectra are very similar to those of compounds **35** and **36** and independent of chain length, confirming that the convergence limit of ECL is reached in the shortest molecule. The solution spectra of compounds **37** are practically identical to that of **36c**, indicating that even without alkyl spacer, the electronic interactions between the π‐conjugated donor chain and the PBI acceptor are negligible in **36c**, probably because of a large twist angle around the connecting bond due to steric interactions. On the other hand, the absorption spectrum of films of compounds **37** shows the intensity of a band at 470 nm probably due to that aggregation is maximal for compound **37b**, such a feature is generally associated with J‐aggregation and correlated with improved photovoltaic efficiency.^122^ As shown in **Figure** **8**, dark–bright stripes are clearly present in the films of compounds **37** with the periods of 14.2–0.4, 15.0–1.0, and 14.6–0.7 nm for **37a**, **37b**, and **37c**, respectively. These values are identical to those observed in powder small angle X‐ray scattering (SAXS), indicating the formation of lamellar nanostructures in thin films. Selected area electron diffraction (SAED) shows multidiffraction rings, and two of them with the *d*‐spacings of 3.5 and 4.4 Å were attributed to the π−π stacking distances of the adjacent PBI units and conjugated segments, respectively. This indicates that D and A segments segregate each other and form nanostructured films comprising D and A domains. The photovoltaic properties of the oligomers have been characterized with the same type of device as for compounds **35** and **36** with in particular the insertion of a HBL and a LiF layer between the photoactive layer and the Al electrode. As shown in Table 2, the three compounds lead to rather comparable results with PCE values in the range of 2.0%, consistent with the EQE responses. Compound **37b** leads to the best results with a *J*_sc_ of 4.82 mA cm^−2^ and a PCE of 2.33%. Charge mobilities were measured using the space‐charge limited current method on hole‐only and electron‐only devices. The highest hole mobility of 2.5 × 10^−5^ cm^2^ V^−1^ s^−1^ was obtained with compound **37b**, which is consistent with the highest FF value and with the maximum intensity of the aggregation peak in the optical spectrum. On the other hand, all compounds **37** show higher electron mobilities than compound **36c**, which suggests that decoupling PBI from the conjugated donor chain allows a better packing of the PBI units, in agreement with X‐ray diffraction results. In order to extend the absorption of solar light toward longer wavelengths, the authors have introduced a dithienyl benzothiadiazole electron‐acceptor group in the conjugated system of compound **37b** (**38**). This modification enhances the absorption in the 500–600 nm region and results in a further improvement of performances with a *J*_sc_ of 5.32 mA cm^−2^ and PCE of 2.70% which is until now, one of the highest values reported for a SMOSC based on a molecular active material.^121^

**Scheme 11:**
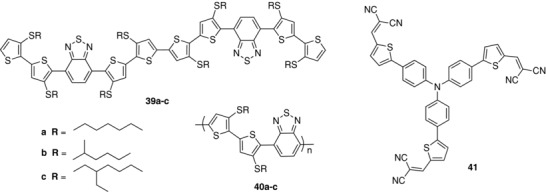
Chemical structure of compounds **39**–**41**.

**Figure 9:**
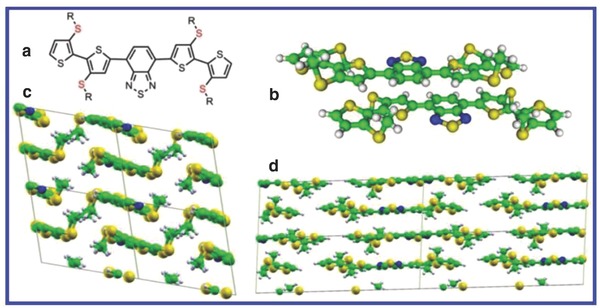
a) Molecular structure of the model pentamer model employed for DT calculations. b) Dimer and c,d) two different views of the 3D periodic crystal structure. Reproduced with permission.^123^ Copyright 2018, American Chemical Society.

Di Maria et al. have synthesized low bandgap decamers and polymers combining 3‐alkylsulfanylthiophenes and benzothiadiazole^123^ with the purpose of investigating the effects of introduction of additional sulfur in the structure (**Scheme** **11**). Two series of compounds substituted by linear and branched substituents were synthesized in good yields. In solution, decamers **39** present absorption maxima in the 510–520 nm region, while the polymers absorb at longer wavelengths (from 538 nm for **40a** up to 612 nm for **40c**). As expected, solution‐cast films absorb at longer wavelengths ≈ 560 nm for the decamers and from 614 to 672 nm for the polymers. On the basis of previous results and density functional theory (DFT) calculations (**Figure** **9**), it was proposed that the molecules adopt a coplanar conformation with thioalkyl substituents perpendicular to the conjugated chains. The various materials have been evaluated in SMOSCs of simple structure ITO/PEDOT:PSS/**39** or **40**/Al. As shown in Table 2, the best results were obtained with decamer **39c** with ethylhexyl substituents which give a *J*_sc_ of 2.36 mA cm^−2^ and a PCE of 0.54%. This compound also gives the best results when mixed with PC_61_BM in conventional BHJ (PCE = 1.80%).^123^

Examination of the above discussed various molecular D–A structures shows that besides the variety of D and A units, these compounds differ by the degree of electronic communication of D and A between fully isolated D and A in the case of flexible linkers, to partially or even fully conjugated structures, as in **33**–**41**. Thus, in **34**, the conjugation between the D and A is limited by the pyrrolidone bridge. In **35**, it is controlled by the departure from planarity caused by steric interactions between the PBI block and the adjacent fluorene. However, together with the previously reported star‐shaped triphenylamine‐based molecule **41**,^31^, ^40^ oligomers **40** are until now the only fully conjugated D–A structures, leading to SMOSCs with significant photocurrents (*J*_sc_ = 1.70–2.40 mA cm^−2^). It is rather surprising that such systems in which geminate recombination is expected to be very high can nevertheless present significant photovoltaic activity. These results pose several intriguing questions regarding in particular the minimal size of a D–A system capable to dissociate excitons, the structural factors which control charge separation/recombination, as well as a molecular packing compatible with the formation of independent pathways for the transport of electrons and holes. Such questions have a link with the problems posed by the use of highly dipolar D–A molecules as donor material in two‐component OPV cells. According to the Bässler model,^124^, ^125^ only compounds devoid of dipole moment are expected to exhibit efficient charge‐carrier transport because the energetic disorder associated with dipole moments is thought to impede charge hopping. This view has been questioned by Würthner and co‐workers who showed that some push–pull cyanines that form centrosymmetric dimers with perfectly canceled dipole moments in the solid state can anyway lead to relatively high PCE.^126^ This suggests that further investigations of these questions will certainly provide valuable information regarding the judicious use of strongly dipolar molecules for the design of D–A materials for SMOCSs.

A large part of SMOSCs fabricated with molecular donors has involved fullerene–donor dyads. Besides simple D–L–A dyads (where L denotes a nonconjugating linker), other architectures such as A–L–D*–*L–A reminiscent of the structure of advanced molecular donors have been described.^36^ The introduction of acceptor units in such donors creates an internal charge transfer which probably contributes to limit charge recombination with the attached fullerene. The promising results obtained with some small molecular dyads with partially conjugated D and A confirm that the BHJ model is not the unique route toward efficient SMOSCs. Finally, the fact that some fully conjugated molecular D–A systems can produce significant photocurrents pose intriguing fundamental questions and this type of materials certainly deserve further experimental and theoretical work.

## 4 Conclusions and Perspectives

Recent advances in the synthesis of SMOCS materials and the PCE of 4.0–5.0% recently reported definitely demonstrate that SMOSCs are not doomed to low efficiency and that further progress can be expected in a near future. Furthermore, the stabilization of the morphology of the active layer, which remains one of the major problems of two‐component BHJs has been demonstrated in several works. For historical reasons, “double‐cable” polymers with fullerene acceptors have been the most widely investigated model of active material for SMOSC and the PCE of 5.58% recently reported for a cell based on P3HT and C_60_ equals the best results obtained with two‐component cells. Considerable effort has been invested in the synthesis of D–A block copolymers. These materials generally require complex syntheses and thorough purifications. However, except for a reported PCE of 3.0%, which remains an isolated result,^84^ the efficiency of SMOSCs based on these materials remain modest. On the other hand, the high PCE (≈4.0%) recently obtained with a fully conjugated D–A polymer based on advanced building blocks^92^ can be expected to stimulate further work in this direction.

Besides complex dyads, triads, or extended oligomers, recent work has shown that rather simple molecular D–A structures can lead to highly efficient SMOSCs and PCEs of ≈2.50–2.70% have been recently reported. In view of the rudimentary level of understanding of the structural factors involved in the design of SMOSC materials, molecular systems are probably more appropriate for analyzing such parameters and to progress toward the definition of relevant synthetic principles. To this end, further simplifications of model D–A systems and the analysis of questions such as the minimal extension of the D and A blocks and their degree of intramolecular interactions in the ground and excited states clearly require further work.

**Figure 10:**
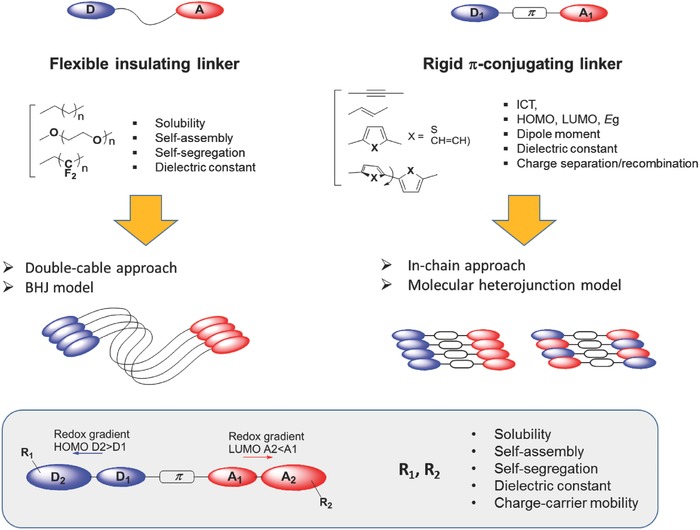
Synthetic parameters for the design of photoactive materials for SMOSCs.

Taking into account recent advances in SMOSC materials as well as progress in the synthesis of highly efficient molecular donors and acceptors, future research on the chemistry of SMOSC material could take into consideration the following points:

1) *Choice of D and A Blocks*: From the beginning, the design active material for SMOSC has followed the state of the art of two‐component BHJ cells. Thus, PPV derivatives have been replaced by P3HT and then by low bandgap polymers, whereas early molecular donor blocks of homogeneous structure such as conjugated oligomers or acenes have been recently replaced by hybrid donor systems with internal charge transfer (ICT). Similarly, the C_60_‐based acceptors used in early SMOSCs have been progressively replaced by C_70_ derivatives and more recently by simple nonfullerene acceptors such as NDI and PBI. The intense research effort invested in the synthesis of OPV materials in the past decade has generated a vast library of polymeric and molecular donors and acceptors which will allow the synthesis of many complementary D–A combinations with optimized coverage of the solar spectrum and controlled energy levels. In particular, the new classes of highly efficient molecular donors and nonfullerene acceptors recently developed have not been used for the synthesis of SMOSC materials yet, and it can be expected that these advanced building blocks will have a strong impact on the development of SMOSCs in the near future.
2) *Charge‐Carrier Mobility*: The optimization of the charge‐carrier mobility remains one of the key problems of the design of OPV materials. Although some publications on SMOSCs have reported mobility measurements, such data are often lacking and further effort in the analysis and optimization of the processes of charge transport in SMOCSs appears necessary. Indeed, a database presenting the values of the ratio of hole and electron mobilities in the various classes of SMOSC materials will be certainly helpful for future design of new materials.
3) *Charge Stoichiometry*: It is generally accepted that balanced hole and electron mobilities are required for optimal PCE. However the potential stoichiometry of positive and negative charges in two‐component BHJs or in a D–A system for SMOSC is seldom considered. Cyclic voltammetry is often used to estimate the highest occupied molecular orbital (HOMO) and lowest unoccuppied molecular orbital (LUMO) level of the compounds from the onset of their oxidation and reduction processes. However, more detailed electrochemical studies could be helpful. In particular, the determination of the number and energy levels of the eventual upper oxidation and reduction states and of their energy difference with the first oxidation and reduction steps generally involved in charge transport can provide valuable information for molecular engineering. For example, in highly conjugated systems, a direct two‐electron oxidation leads to the formation of bipolarons which are not expected to contribute to charge transport. Consequently, when the first and second oxidation (or reduction) steps are very close in energy, the passage from solution to the solid state can further reduce this energy difference and hence promote the direct formation of bipolarons.
4) *Dielectric Constant*: The low dielectric constant of organic semiconductors remains one of the major limitations of OPV cells. Increasing the dielectric constant of active materials can contribute to lower the energy of exciton splitting and limit charge recombination. Although this question has been discussed,^12^, ^13^, ^127^ work in this direction remains scarce.^128^, ^129^, ^130^ The introduction of polyether or cyanoalkyl groups in the donor structure has been shown to significantly increase the dielectric constant.^128^, ^129^, ^130^ Other possible strategies involve the increase of the dipole moment of D and/or A units,^110^ or the development of hyperbranched molecules.^127^
5) *Connection of D and A*: The linker L connecting D and A blocks is one of the major component of SMOSC materials. As already discussed, the length and chemical composition of L determines two limiting cases (**Figure** **10**). In the “double‐cable” models, L is an insulating flexible chain of sufficient length to allow separated self‐organization of D and A. In the molecular heterojunction model, the intramolecular electronic interactions between D and A are controlled by the conjugation efficiency of L which leads to different situations from weakly interacting D and A in the case of a methylene bridge, to increasingly stronger interactions with more efficient bridges such as acetylenic, aromatic, olefinic, and finally direct connection of D and A. In this latter case, the steric control of the dihedral angle between D and A constitutes an interesting approach for a fine‐tuning of D–A interactions. These interactions determine the strength of the ICT and hence the energy levels, light‐harvesting properties, and dipole moment of the D–A architecture. Furthermore, the modulation of the dipole moment by the conjugating linker will also influence intermolecular interactions and hence the packing arrangement of the molecules in the material (head‐to‐tail or cofacial). Consequently, the optimization of the conjugating bridge is clearly one of the keys of the efficiency of D–π–A systems. Although efficient SMOSCs based on conjugated D–A systems have been reported, the structural factors involved in charge separation remain poorly understood. The fact that such systems can produce significant photocurrent is counterintuitive and suggests a possible equilibrium between the charge recombination and charge transport. In this regard, the creation of a redox gradient in D and A around the connecting point and the introduction of substituents (R_1_, R_2_) for improving hole and electron mobilities could contribute to limit charge recombination.

Although research on OPV research was initially motivated by the development of a low cost and environmentally friendly complement, or even alternative to silicon solar cells, the considerable improvement of the performances of OPV cells has generated an increasing complexification of the synthesis of active materials. The “OPV paradox” is that the continuous increase of PCE does not open the door to industrialization but on the contrary tend to relegate OPV into a purely academic topic. This situation can be explained by an excessive optimism and by a quasi‐exclusive focus of research on PCE while neglecting issues such as the cost, environmental impact, scalability, and stability of materials and devices.^131^, ^132^, ^133^, ^134^ Although these questions deserve specific research efforts, economic considerations cannot be the exclusive motivation of research and the multiple fundamental problems still posed by the physics of OPV and by the chemistry of active materials clearly require a continuation of research effort.

SMOSCs are the ultimate stage of simplification of OPV cells and represent a fascinating target with considerable technological and fundamental potential implications. Although many initial prototypes of SMOCS showed only modest efficiencies, advances in the synthesis of donor and acceptor materials for BHJs has contributed to spectacular progress with PCE now in the range 5.0%. The synthesis of new molecular architectures leading to highly efficient SMOSCs is certainly a risky and ambitious challenge. However, recent advances in the chemistry of OPV materials and the expertise accumulated during two decades on related topics such as molecular heterojunctions, DSSCs, liquid crystals, or TTF chemistry provide a general context propitious to a real and successful take‐off of research on SMOSCs.

## Conflict of Interest

The authors declare no conflict of interest.
